# Optimal Doses of Specific Antipsychotics for Relapse Prevention in a Nationwide Cohort of Patients with Schizophrenia

**DOI:** 10.1093/schbul/sbac039

**Published:** 2022-05-07

**Authors:** Heidi Taipale, Antti Tanskanen, Jurjen J Luykx, Marco Solmi, Stefan Leucht, Christoph U Correll, Jari Tiihonen

**Affiliations:** Department of Forensic Psychiatry, University of Eastern Finland, Niuvanniemi Hospital, Kuopio, Finland; Department of Clinical Neuroscience, Karolinska Institutet, Stockholm, Sweden; School of Pharmacy, University of Eastern Finland, Kuopio, Finland; Center for Psychiatry Research, Stockholm City Council , Stockholm, Sweden; Department of Forensic Psychiatry, University of Eastern Finland, Niuvanniemi Hospital, Kuopio, Finland; Department of Clinical Neuroscience, Karolinska Institutet, Stockholm, Sweden; Center for Psychiatry Research, Stockholm City Council , Stockholm, Sweden; Population Health Unit, Finnish Institute for Health and Welfare, Helsinki, Finland; Department of Psychiatry, UMC Utrecht Brain Center, University Medical Center Utrecht, Utrecht University, Utrecht, The Netherlands; Department of Translational Neuroscience, UMC Utrecht Brain Center, University Medical Center Utrecht, Utrecht University, Utrecht, The Netherlands; GGNet Mental Health, Warnsveld, The Netherlands; Department of Psychiatry, University of Ottawa, Ottawa, ON, Canada; Department of Mental Health, The Ottawa Hospital, Ottawa, ON, Canada; Ottawa Hospital Research Institute (OHRI), Clinical Epidemiology Program, University of Ottawa, Ottawa, ON, Canada; Section Evidence Based Medicine in Psychiatry and Psychotherapy, Department of Psychiatry and Psychotherapy, Klinikum rechts der Isar, Technical University of Munich, München, Germany; Department of Psychiatry, The Zucker Hillside Hospital, Northwell Health, Glen Oaks, NY, USA; Department of Psychiatry and Molecular Medicine, Donald and Barbara Zucker School of Medicine at Hofstra/Northwell, Hempstead, NY, USA; Department of Child and Adolescent Psychiatry, Charité Universitätsmedizin Berlin, Berlin, Germany; Department of Forensic Psychiatry, University of Eastern Finland, Niuvanniemi Hospital, Kuopio, Finland; Department of Forensic Psychiatry, University of Eastern Finland, Niuvanniemi Hospital, Kuopio, Finland; Department of Clinical Neuroscience, Karolinska Institutet, Stockholm, Sweden; Center for Psychiatry Research, Stockholm City Council , Stockholm, Sweden; Neuroscience Center, University of Helsinki, Helsinki, Finland

**Keywords:** psychosis, prevention, hospitalization

## Abstract

**Background and Hypothesis:**

Optimal doses of most antipsychotics in the maintenance treatment of schizophrenia are unknown. We aimed to study the risk of severe relapse indicated by rehospitalization for different dose categories of 15 most frequently used antipsychotics in monotherapy in Finland.

**Study Methods:**

We studied the risk of rehospitalization (Adjusted Hazard Ratio, aHR) associated with six antipsychotic monotherapy dose categories (as time-varying dose, measured in defined daily dose, DDDs/day) in a nationwide cohort of persons diagnosed with schizophrenia (*n* = 61 889), using within-individual analyses to eliminate selection bias.

**Study Results:**

Among the 15 most widely used antipsychotics, 13 had a U- or J-shaped dose-response curve, showing the lowest risks of relapse for doses of 0.6–<1.1 DDDs/day vs nonuse of antipsychotics. The exceptions were oral perphenazine (aHR = 0.72, 95% CI = 0.68–0.76, <0.6 DDDs/day), and olanzapine-long-acting injectable (LAI), which had the lowest aHR of any antipsychotic (aHR = 0.17, 95% CI = 0.11–0.25, 1.4–<1.6 DDDs/day). Certain risperidone and perphenazine doses <0.9 DDD/day were associated with 21%–45% lower risk of rehospitalization (*P* < .001) than the standard dose of 0.9–1.1 DDD/day (ie, 5 mg for risperidone and 30 mg for perphenazine).

**Conclusions:**

For most antipsychotics, the risk of severe relapse was the lowest during use of standard dose. Our results suggest that olanzapine LAI is highly effective in dose ranges >0.9 DDD/day, and especially at 1.4–<1.6 DDDs/day (405 mg/4 weeks) associated with substantially lower risk of rehospitalization than any dose of any other antipsychotic. The current WHO standard dose definitions appear to be clearly too high for perphenazine and somewhat too high for risperidone.

## Introduction

Most patients with schizophrenia respond well to antipsychotic treatment in the acute phase of illness,^1^ and the main challenge is to prevent relapses by using maintenance treatment.^2^ The optimal antipsychotic dose has been studied in several reviews and meta-analyses,^3–7^ concluding that no additional benefit can be achieved above standard doses (corresponding to 1 defined daily dose [DDD]/day, WHO, see Supplementary Table 1), but doses below standard dose are associated with an increased relapse risk.^6,7^ A recent study on optimal antipsychotic doses in a nationwide incident cohort showed that a standard dose corresponding to 0.9–1.1 DDD/day was associated with the lowest risk of rehospitalization.^8^ However, no studies have investigated different dose categories of specific antipsychotics in different formulations, which is an important unmet need as optimal doses may differ across antipsychotics and formulations. We aimed to study risks of severe relapse indicated by rehospitalization for different dose categories of 15 most frequently used antipsychotics, by using prospectively gathered data including all patients with schizophrenia in Finland. The primary analysis was conducted in within-individual design where each patient serves as his/her own control to eliminate selection bias. We hypothesized that standard dose of each antipsychotic is associated with the best outcome.

## Methods

The cohort was identified from nationwide Hospital Discharge register and included all persons hospitalized due to schizophrenia during 1972–2014 in Finland and who were alive at January 1, 1996 (*N* = 61 889). Schizophrenia was defined as International Classification of Diseases (ICD) version 10 codes F20 and F25 (corresponding ICD-9 and ICD-8 code 295*). Data were linked via personal identification numbers on all inpatient stays (dates and diagnoses 1972, from Hospital Discharge register), dispensed medications (from Prescription register 1995–2017), and deaths (causes of death register 1972–2017). Observation time for medication use started on January 1, 1995 when the Prescription Register was established. We identified the first-episode schizophrenia cohort as persons who had their first diagnosis of schizophrenia during 1996–2014, without a previous diagnosis and who had not used antipsychotics in the one year before their first diagnosis (to ensure they are incident cases). Concerning patients with first-episode schizophrenia, the follow-up started on January 1, 1996 for those diagnosed before that date, and on the date of diagnosis for those diagnosed later, and ended at death or December 31, 2017. Patients who died between 1972 and 1996 were not included, as we could not follow their medication use. Relapse was defined as rehospitalization based on the main diagnosis of F20–F29. Relapse was treated as a recurrent event, ie possible to happen multiple times for the same person.

Antipsychotic use was defined as Anatomical Therapeutic Chemical (ATC) classification code N05A excluding lithium. Antipsychotic dispensings were derived from the Prescription register, and included ATC code, date of dispensing, product information (name of drug product, strength, package size), the number of packages dispensed, and dispensed amount in defined daily doses (DDDs) as defined by the WHO (WHO DDD).^9^ Prescribed doses are not available in the Prescription register. Episodes when antipsychotic use started and ended, were modeled with the PRE2DUP method.^10^ The method is based on the mathematical modeling of personal drug purchasing behavior from the dispensing data. By utilizing information on dispensing dates and amounts in DDDs, together with drug package level information and expert-opinion derived control parameters, the method constructs antipsychotic use episodes with start and end dates. Irregularities in dispensing events, caused by eg stockpiling and time periods spent in hospital care when drugs are provided by the unit treating and not recorded in the Prescription register, are taken into account.^10,11^ Each ATC code and by separating oral and long-acting injectable antipsychotic (LAI) formulations were modeled separately.

After formation of antipsychotic use episodes, we calculated temporal dose estimates at each dispensing date as the sum of the dispensed DDD amount of the two previous dispensings divided by the outpatient time of these dispensings. This temporal dose estimate was expressed as DDDs/day dose. The dose estimate was valid until the next dispensing when the dose was reevaluated (ie dose changes were possible only at dispensing dates). Antipsychotic episodes including three or more dispensings were processed by considering dispensings in a stepwise manner, starting from the second dispensing and proceeding till the last dispensing. For antipsychotic episodes including only one or two dispensings, dose estimates were calculated as dispensed DDDs divided by outpatient time (ie, no dose changes were possible within these short episodes).

Antipsychotic episodes were subdivided into time periods when a certain dose (as DDDs/day) was consistently used in the following categories: <0.6, 0.6–<0.9, 0.9–<1.1, 1.1–<1.4, 1.4–<1.6, ≥1.6. Categories were formed around most frequent clinically relevant doses (0.5, 1.0, and 1.5 DDDs/day) with small variation and inaccuracy allowed (ie 0.9–<1.1 describes 1.0 DDDs/day use). We focused on antipsychotic monotherapy only and excluded time periods when more than one antipsychotic was used at the same time (ie antipsychotic polytherapy). A person may contribute person-time to multiple or even all dose categories of a drug or of multiple antipsychotic drugs used in monotherapy during the follow-up (Supplementary Figure 1).

## Statistical Analyses

The fifteen most common antipsychotic monotherapies were analyzed on drug and dose category levels. Time periods of specific dose categories were compared with time periods of nonuse of any antipsychotics for relapse risk. In sensitivity analyses, the reference was the most commonly used drug and dose category, being oral olanzapine >1.6 DDDs/day in this cohort (most common in terms of both the number of users and person-years of use). In addition, sensitivity analyses where doses of each specific antipsychotic were compared with standard dose of that antipsychotic.

To reduce chances of confounding by indication, the main analyses were conducted in a within-individual design where all comparisons are conducted within the same individual, ie, each individual acts as his or her own control in a stratified Cox model.^12^ Time was reset to zero after each relapse. The impact of time-invariant factors, such as genetics and initial severity of the illness, are eliminated by the design, and we adjusted for time-varying factors, which were time since cohort entry, temporal order of specific antipsychotics, and use of antidepressants, mood stabilizers, benzodiazepines, and related drugs. Sensitivity analyses were conducted by censoring the first 30 days from all dose categories, as the full therapeutic effect is usually not reached immediately, especially when the dose has to be titrated up slowly. In these analyses, censoring was equally applied to nonuse periods. Sensitivity analyses also included analyses stratified by baseline age (≤45 vs >45 years), schizoaffective diagnoses (where schizoaffective disorder was defined as ICD-10 F25 and schizophrenia F20), and among first-episode patients.

As only persons with variation in exposure and outcome event (relapse) contribute to the within-individual comparisons, also traditional between-individual Cox models including all patients were conducted. Between-individual models were adjusted for age, sex, temporal order of treatment, previous number of psychiatric rehospitalizations, calendar year, use of antidepressants, benzodiazepines, and related drugs, anticholinergic antiparkinson drugs and statins, diagnosis of cardiovascular disease, diabetes, cancer, asthma/ COPD, substance abuse, suicide attempt, liver disease, and renal disease. The results are reported as adjusted HRs (aHRs) with 95% confidence intervals (CIs). The significance of differences between aHRs was tested by comparing two hazard ratios from their betas and standard errors as computed by a Cox model, and tested by Student *t* test for dependent samples (R-package survcomp, function hr.comp, version 1.44).

Permissions for this study were granted by pertinent institutional authorities at the National Institute for Health and Welfare of Finland, The Social Insurance Institution of Finland, and Statistics Finland. The funders of the study had no role in study design, data collection, data analysis, data interpretation, or writing of the report.

## Results

The mean age of the cohort was 46.7 years (SD 16.0) at the start of follow-up, they had spent a mean of 8.8 years (SD 9.0) since their first schizophrenia diagnosis, and 50.3% (*N* = 31 104) were men. Oral olanzapine was the most commonly used antipsychotic (16 131 users), followed by risperidone (*N* = 13 083), clozapine (*N* = 11 828), and quetiapine (*N* = 10 838). Of specific dose categories of specific antipsychotic monotherapies, the most commonly used antipsychotic was oral olanzapine ≥1.6 DDDs/day, followed by risperidone <0.6 DDDs/day (table 1). Distribution of used doses varied between specific antipsychotics. All dose categories of clozapine were used relatively frequently. Oral olanzapine was more commonly used with ≥1.6 DDDs/day dose (*N* = 11 351 users vs *N* = 7365 users of standard dose) whereas quetiapine and risperidone were more commonly used <0.6 DDDs/day dose than as standard dose (table 1).

**Table 1.** The Risk of Relapse Associated with Specific Dose Categories (in DDDs Per Day) of Specific Antipsychotic Monotherapies, in Rank Order, Compared with Nonuse of Antipsychotics. Information on *N* of Users, Person-years (PYs), and Events (Rehospitalizations) also Provided

Drug	Dose	aHR (95% CI)	*P*-value	Users	PYs	Events
Olanzapine LAI	1.4–<1.6	0.17 (0.11–0.25)	<.0001	376	268	34
Olanzapine LAI	0.9–<1.1	0.21 (0.13–0.35)	<.0001	233	147	21
Olanzapine LAI	1.1–<1.4	0.24 (0.17–0.36)	<.0001	352	214	35
Olanzapine LAI	≥1.6	0.36 (0.32–0.42)	<.0001	727	823	315
Risperidone LAI	0.9–<1.1	0.37 (0.33–0.41)	<.0001	2016	2706	408
Clozapine	0.9–<1.1	0.37 (0.36–0.39)	<.0001	8005	12 967	2600
Clozapine	1.1–<1.4	0.39 (0.37–0.41)	<.0001	8811	17 713	3924
Olanzapine	0.9–<1.1	0.40 (0.38–0.43)	<.0001	7365	12 111	1464
Clozapine	0.6–<0.9	0.41 (0.39–0.43)	<.0001	8004	15 546	3436
Zuclopenthixol LAI	<0.6	0.42 (0.37–0.47)	<.0001	1494	4278	507
Perphenazine LAI	0.6–<0.9	0.42 (0.38–0.46)	<.0001	1900	3365	672
Zuclopenthixol LAI	0.6–<0.9	0.42 (0.39–0.47)	<.0001	1968	3951	736
Haloperidol LAI	0.9–<1.1	0.44 (0.38–0.52)	<.0001	778	1225	251
Perphenazine LAI	<0.6	0.44 (0.39–0.49)	<.0001	1523	3872	579
Risperidone LAI	0.6–<0.9	0.44 (0.40–0.49)	<.0001	2366	3402	653
Risperidone LAI	1.1–<1.4	0.44 (0.40–0.49)	<.0001	2008	2709	612
Haloperidol LAI	1.4–<1.6	0.45 (0.36–0.55)	<.0001	541	475	128
Risperidone LAI	<0.6	0.45 (0.38–0.54)	<.0001	1056	852	190
Olanzapine	1.4–<1.6	0.45 (0.41–0.48)	<.0001	6064	6417	1159
Zuclopenthixol	0.9–<1.1	0.46 (0.35–0.61)	<.0001	417	425	80
Zuclopenthixol	0.6–<0.9	0.46 (0.38–0.56)	<.0001	667	1071	162
Perphenazine LAI	1.1–<1.4	0.46 (0.41–0.52)	<.0001	1480	1463	401
Clozapine	1.4–<1.6	0.46 (0.44–0.49)	<.0001	7222	6981	2169
Perphenazine LAI	0.9–<1.1	0.47 (0.41–0.53)	<.0001	1378	1773	395
Zuclopenthixol LAI	0.9–<1.1	0.47 (0.43–0.52)	<.0001	1915	2978	610
Haloperidol LAI	<0.6	0.48 (0.40–0.58)	<.0001	599	1683	187
Clozapine	<0.6	0.50 (0.47–0.53)	<.0001	5957	10 502	2665
Haloperidol LAI	0.6–<0.9	0.51 (0.43–0.61)	<.0001	764	1194	226
Aripiprazole	0.9–<1.1	0.51 (0.44–0.60)	<.0001	1639	1735	249
Olanzapine	0.6–<0.9	0.52 (0.49–0.56)	<.0001	7392	9741	1775
Aripiprazole	<0.6	0.53 (0.43–0.66)	<.0001	842	807	155
Olanzapine	<0.6	0.53 (0.49–0.57)	<.0001	6224	10 122	1536
Clozapine	≥1.6	0.53 (0.51–0.55)	<.0001	8190	19 349	6664
Zuclopenthixol LAI	1.4–<1.6	0.54 (0.47–0.62)	<.0001	997	646	299
Risperidone	0.6–<0.9	0.54 (0.51–0.58)	<.0001	5920	9224	1728
Perphenazine LAI	1.4–<1.6	0.56 (0.46–0.68)	<.0001	725	398	150
Aripiprazole	0.6–<0.9	0.56 (0.47–0.66)	<.0001	1280	1056	218
Zuclopenthixol LAI	1.1–<1.4	0.56 (0.51–0.62)	<.0001	1531	1542	615
Olanzapine	1.1–<1.4	0.57 (0.54–0.61)	<.0001	7498	5979	1615
Haloperidol LAI	1.1–<1.4	0.58 (0.50–0.67)	<.0001	786	924	277
Olanzapine	≥1.6	0.58 (0.56–0.60)	<.0001	11 351	26 988	7598
Chlorprotixene	0.6–<0.9	0.60 (0.53–0.69)	<.0001	1598	2537	361
Zuclopenthixol LAI	≥1.6	0.63 (0.59–0.67)	<.0001	1919	2957	1608
Levomepromazine	0.9–<1.1	0.64 (0.47–0.86)	.0035	423	328	79
Zuclopenthixol	1.1–<1.4	0.64 (0.50–0.82)	.0004	416	309	117
Haloperidol LAI	≥1.6	0.64 (0.58–0.70)	<.0001	1043	1853	899
Risperidone	<0.6	0.64 (0.61–0.67)	<.0001	9695	22 175	3773
Haloperidol	0.9–<1.1	0.65 (0.54–0.78)	<.0001	562	576	186
Zuclopenthixol	≥1.6	0.66 (0.55–0.78)	<.0001	580	962	275
Haloperidol	0.6–<0.9	0.66 (0.57–0.77)	0.0827	829	767	292
Risperidone	1.1–<1.4	0.66 (0.60–0.72)	<.0001	2889	3098	858
Olanzapine LAI	0.6–<0.9	0.67 (0.43–1.05)	0.0827	136	83	32
Zuclopenthixol	<0.6	0.67 (0.59–0.77)	<.0001	1076	2307	383
Aripiprazole	1.4–<1.6	0.68 (0.52–0.89)	0.0051	597	254	84
Haloperidol	1.1–<1.4	0.68 (0.56–0.82)	<.0001	502	335	165
Levomepromazine	0.6–<0.9	0.68 (0.57–0.81)	<.0001	813	864	217
Risperidone	0.9–<1.1	0.68 (0.62–0.74)	<.0001	3034	1847	709
Haloperidol	<0.6	0.69 (0.63–0.75)	<.0001	2876	6505	1078
Perphenazine LAI	≥1.6	0.70 (0.63–0.79)	<.0001	1112	1262	586
Quetiapine	0.9–<1.1	0.71 (0.64–0.79)	<.0001	2787	2564	699
Zuclopenthixol	1.4–<1.6	0.72 (0.50–1.04)	.0759	263	147	47
Chlorprotixene	0.9–<1.1	0.72 (0.62–0.85)	.0001	982	1105	234
Perphenazine	<0.6	0.72 (0.68–0.76)	<.0001	6761	21 906	3169
Quetiapine	1.1–<1.4	0.73 (0.67–0.81)	<.0001	3048	2134	741
Chlorprotixene	<0.6	0.73 (0.68–0.79)	<.0001	3998	10 223	1349
Quetiapine	1.4–<1.6	0.74 (0.67–0.83)	<.0001	2354	2002	557
Chlorprotixene	1.1–<1.4	0.75 (0.63–0.90)	.0015	915	847	205
Levomepromazine	<0.6	0.75 (0.71–0.80)	<.0001	5114	8584	1742
Levomepromazine	1.1–<1.4	0.77 (0.56–1.06)	.1032	364	268	72
Haloperidol	1.4–<1.6	0.77 (0.58–1.01)	.0604	318	227	79
Risperidone LAI	1.4–<1.6	0.77 (0.65–0.91)	.0021	1024	384	225
Aripiprazole	≥1.6	0.78 (0.69–0.89)	.0002	1489	1351	487
Chlorprotixene	1.4–<1.6	0.79 (0.61–1.03)	.0771	474	254	88
Quetiapine	0.6–<0.9	0.79 (0.73–0.86)	.828	3867	3560	1040
Quetiapine	<0.6	0.79 (0.75–0.84)	.9391	7633	12 081	2510
Perphenazine	0.6–<0.9	0.83 (0.74–0.93)	.0008	1823	2270	584
Aripiprazole	1.1–<1.4	0.87 (0.73–1.05)	.1401	1214	554	205
Risperidone	1.4–<1.6	0.94 (0.80–1.09)	.3805	1317	621	291
Olanzapine LAI	<0.6	0.95 (0.34–2.68)	.9284	26	12	8
Quetiapine	≥1.6	0.99 (0.94–1.05)	.828	4295	4559	2484
Levomepromazine	≥1.6	1.01 (0.77–1.33)	.9391	385	179	103
Chlorprotixene	≥1.6	1.03 (0.88–1.20)	.7097	908	630	332
Haloperidol	≥1.6	1.07 (0.92–1.25)	.3574	628	507	321
Levomepromazine	1.4–<1.6	1.11 (0.71–1.75)	.6487	176	80	36
Perphenazine	0.9–<1.1	1.32 (1.09–1.61)	.0053	739	345	188
Risperidone	≥1.6	1.34 (1.22–1.47)	<.0001	2507	1057	869
Risperidone LAI	≥1.6	1.41 (1.25–1.59)	<.0001	1332	401	493
Perphenazine	1.1–<1.4	1.57 (1.21–2.04)	.0006	487	144	120
Perphenazine	1.4–<1.6	2.54 (1.70–3.79)	<.0001	193	27	50
Perphenazine	≥1.6	3.35 (2.37–4.73)	<.0001	560	44	88

*Note:* DDD, defined daily dose; LAI, long-acting injectable antipsychotic; aHR, adjusted Hazard Ratio.

**Fig. 1.**
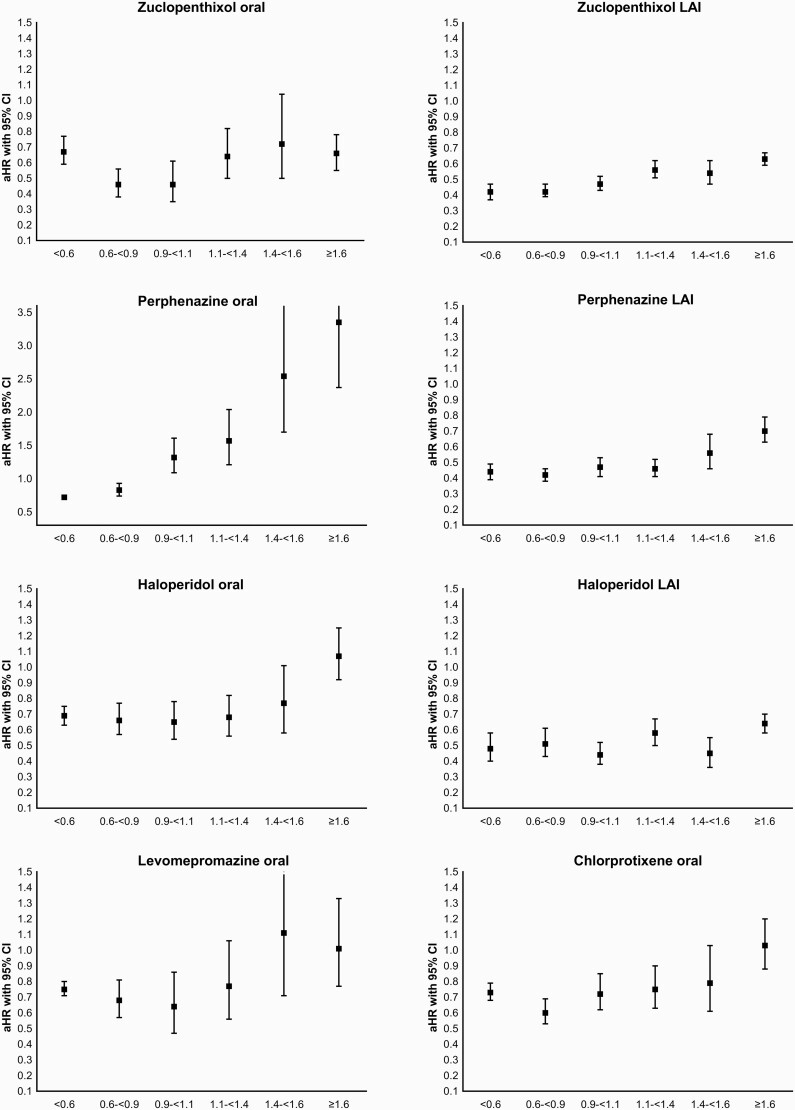

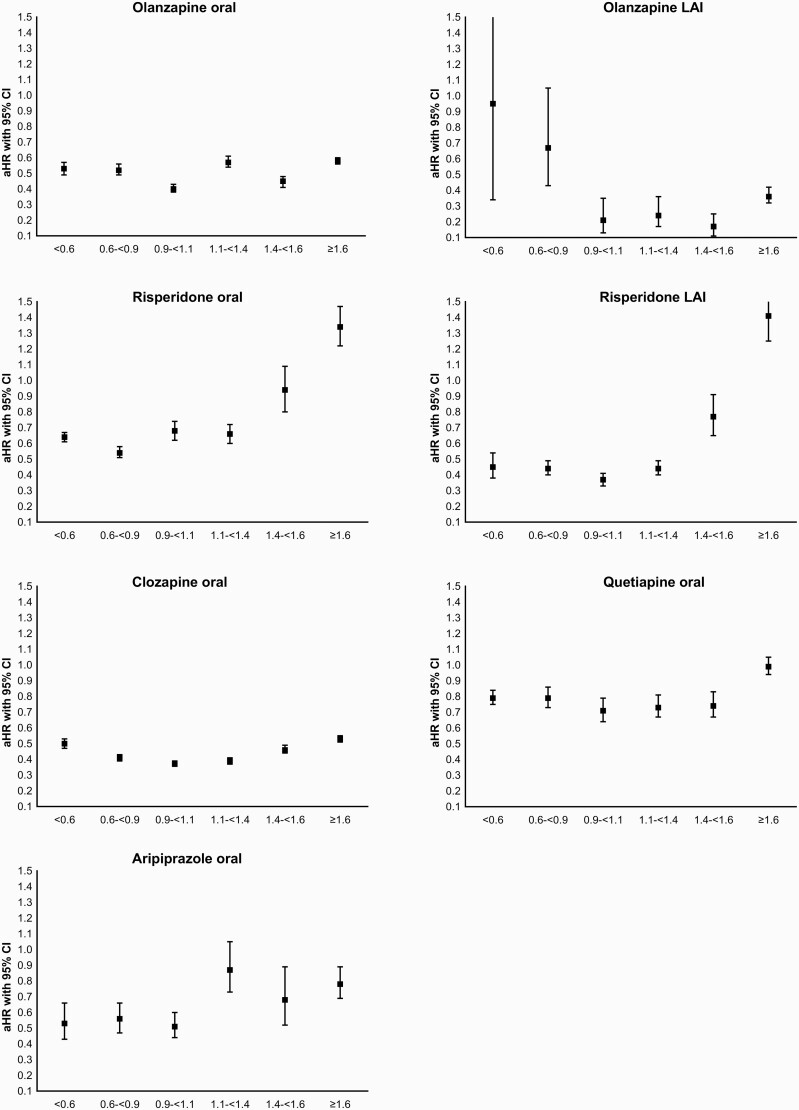
Adjusted Hazard Ratios for 15 most widely used antipsychotics in monotherapy, reference: nonuse of antipsychotics. Note the different scale for perphenazine oral.

Compared to nonuse of antipsychotics within the same individual, 13 out of 15 antipsychotic monotherapies showed a U-shaped or J-shaped dose-response curve, showing the lowest aHR for relapse for standard dose of 0.9–1.1 DDDs/day (nine specific antipsychotics out of 13, namely oral levomepromazine, haloperidol oral and LAI, oral zuclopenthixol, oral clozapine, oral olanzapine, oral quetiapine, risperidone LAI and oral aripiprazole), or 0.6–<0.9 DDDs/day (four out of 13 specific antipsychotics, namely perphenazine LAI, chlorprothixene, zuclopenthixol LAI, oral risperidone) compared with nonuse of antipsychotics (figure 1). The exceptions in dose categories with the lowest HRs were oral perphenazine (aHR = 0.72, 95% CI = 0.68–0.76, <0.6 DDDs/day) and olanzapine LAI (aHR = 0.17, 95% CI = 0.11–0.25, 1.4–<1.6 DDDs/day), the latter of which had the lowest aHR for any antipsychotic (54% lower than for second best, nonolanzapine LAI drug, which was risperidone LAI with a dose of 0.9–1.1 DDDs/day, HR = 0.37, 95% CI = 0.33–0.41), *P* < .0001 for difference. In addition to the dose of 1.4–1.6 DDD/day, all doses of olanzapine LAI above 0.9 DDDs/day were associated with excellent outcomes (aHRs below 0.25). Oral risperidone dose 0.6–0.9 DDDs/day was associated with 21% lower risk of hospitalization compared with standard dose 0.9–1.1 DDD/day (aHR = 0.54, 95% CI = 0.51–0.58 vs aHR = 0.68, 95% CI = 0.62–0.74), *P* < .001 for difference.

High doses (>1.6 DDDs/day, corresponding to >48 mg/day) of oral perphenazine (aHR=3.35, 95% CI = 2.37–4.73), risperidone LAI (>60 mg/2 weeks; aHR 1.41, 95% CI = 1.25–1.59), and oral risperidone (>8 mg/day; aHR = 1.34, 95% CI = 1.22–1.47) were associated with substantially higher risk of rehospitalization due to schizophrenia than nonuse of antipsychotic (table 1). Concerning oral perphenazine, even standard dose (0.9–<1.1 DDDs/day) was associated with higher risk of relapse (aHR = 1.32, 95% CI = 1.09–1.61), than nonuse of antipsychotics. Sensitivity analyses censoring the first 30 days of all dose categories showed similarly shaped dose-response curves (see Supplementary Table 2).

In head-to-head comparison (including only those patients having used standard dose) using standard dose of each antipsychotic as a reference, similar results were observed (Supplementary Table 3). All antipsychotics with dose ≥1.6 DDDs/day were associated with higher risk of relapse than standard dose of the same drug. For most common oral antipsychotics, low dose (<0.6 DDDs/day) of clozapine, olanzapine, and quetiapine were associated with higher risk of relapse whereas no difference was found for low dose risperidone compared with standard dose of the same drug. A statistically significant superior outcome was seen for the lowest doses of perphenazine (37%–46% lower risk of rehospitalization compared with standard dose), and 0.6–0.9 DDD/day dose for risperidone (20% lower risk). Also, head-to-head comparison with the most commonly used antipsychotic and dose, oral olanzapine >1.6 DDD (ie, including only those patients who had used high-dose oral olanzapine), showed very similar results as the primary analysis (Supplementary Table 4).

The superior outcome for olanzapine LAI 1.4–<1.6 DDDs/day was confirmed in the secondary between-individual analyses (aHR = 0.37, 95% CI = 0.21–0.66, being the lowest aHR for rehospitalization) when compared with the most common antipsychotic/dose category combination oral olanzapine >1.6 DDDs/day (Supplementary Table 5).

Sensitivity analysis stratified on the basis of age, schizoaffective diagnosis, and first-episode status were well in line with the primary analysis (see Supplementary Tables 6, 7, and 8). In the first-episode cohort, the results on high-dose perhenazine (aHR = 6.12, 95% CI = 1.17–32.03), risperidone (aHR = 2.00, 95% CI = 1.46–2.73), and olanzapine LAI (aHR = 0.05, 95% CI = 0.01–0.16) were even more extreme than in the primary analysis.

## Discussion

To our knowledge, this is the first real-world study examining relapse risks for different dosages and formulations of maintenance therapy with antipsychotics in individuals with schizophrenia. Overall, our findings indicate that the best outcomes were associated with standard doses (0.9–<1.1 DDDs/day) in nine of the 15 antipsychotics, and with the next lower dose range (0.6–<0.9 DDDs/day) in 4 of the 15 antipsychotics. However, many low doses (<0.6 DDDs/day) and all high doses (≥1.6 DDDs/day) showed higher risk of relapse than standard dose. Consistent with prior meta-analyses of randomized controlled trials, lower^6,7^ doses than these two (0.6–<0.9 and especially 0.9–<1.1) dose levels were found to be overall associated with poorer relapse outcomes. Two exceptions to these findings were detected, ie better outcomes for relatively low-dose oral perphenazine and for high-dose olanzapine LAI. Relative to all other antipsychotics, olanzapine LAI at DDD of 1.4–<1.6 (equivalent to 405 mg/4 weeks) was associated with the best outcomes in primary and secondary analyses.

The most striking finding in this study was that two widely used antipsychotics with strong D_2_-blockade, risperidone, and perphenazine, at >1.6 DDDs/day were associated with 34%–235% higher risk of rehospitalization compared with nonuse of antipsychotics. Concerning perphenazine, even the standard dose of 0.9–1.1 DDD/day, corresponding to 30 mg/day was associated with significantly higher risk of rehospitalization than nonuse of antipsychotics. It is possible that for some patients, the increased risk of relapse during high-dose treatment may be attributable to exceptionally severe symptoms or a recent increase in dose to control the symptoms. The within-subject approach controls much better for such “confounding by indication” than conventional between-subject analyses, but some residual confounding may exist. However, this explanation is unlikely, because this observation was rather specific for perphenazine and risperidone (oral and LAI formulations), while for weaker D_2_-antagonists (eg, olanzapine, clozapine, quetiapine, or aripriprazole) we did not find this association.^13^ These findings could also be in part attributable to breakthrough psychosis^14–16^ during ongoing use, or to rebound psychosis after abrupt discontinuation because of extrapyramidal or other side effects.^13^ Leucht et al^6^ found that in RCTs higher doses than one DDD are associated with more side effects. Thus, patients who receive high doses may not tolerate them, discontinue use, and relapse. Since the vast majority of the prescriptions in Finland are filled for 90 days, hospitalizations are labeled as having happened during the use of the medication in many cases where the medication was discontinued abruptly during this 90-day period. However, a similar dose-response relationship was observed for oral and LAI formulations, which implies that abrupt discontinuation may not be the only or major contributing factor. Another possible explanation for poorer outcome in the higher dose strata is dopamine supersensitivity,^17–21^ as well as that the current reference DDD value^9^ of 30 mg for perphenazine and 5 mg for risperidone may be too high. On the basis of our results, the appropriate standard dose as DDD would be 18 mg or less for perphenazine, and 3–4.5 mg for risperidone. Perphenazine is a very old antipsychotic and its DDD was defined at a time when higher doses were customary. Indeed, a similar trend was found for zuclopenthixol, for which 0.6 < 0.9 DDD were as good as 0.9–<1.1. A major aim of the WHO DDD classification system is to provide a method to study drug consumption, and it is not meant for recommendations for use or judgements about relative efficacy of drugs^9^ – although it is possible that it might have unintentional implications related to these issues among prescribers. If there is a large gap between DDD of a drug vs the actual commonly used dosage, it might be reasonable to adjust the DDD value. Our results showed that the most frequently used dose category for risperidone was <0.6 DDD (less than 3 mg/day), and in our previous study on this nationwide cohort, the median dose for risperidone was 2.8 mg.^22^ If these results were replicated in other countries, it would imply that the DDD for risperidone should be 3 mg instead of 5 mg.

The sensitivity analyses stratified based on age, schizoaffective diagnosis, and first-episode status confirmed the results of primary analysis. The results on high-dose perphenazine and risperidone were even more extreme in the first-episode cohort which is in line with guideline recommendations that first-episode patients need lower doses.^23^ Certain risperidone and perphenazine doses below 0.9 DDD/day were associated with 21%–45% lower risk of re-hospitalization (*P* < .001) than the standard dose of 0.9–1.1 DDD/day. When the first 30 days were omitted from the beginning of all antipsychotic use and nonuse periods, these results remained the same (23%–45% difference), indicating that recent dose increase to standard dose due to putative worsening of the symptoms does not explain the findings. Additional head-to-head analyses comparing doses of each antipsychotic with its own standard dose as reference (including only those patients who had used standard dose), as well as head-to-head comparison with high-dose olanzapine as reference (including only those patients who had used high-dose olanzapine) were well in line with the primary analysis, indicating the robustness of the findings.

Another major finding was that relatively high dose of 1.4–1.6 DDD/day (equaling to 405 mg once in 4 weeks) of olanzapine LAI was associated with substantially (>50%) lower risk of re-hospitalization than any dose of any other antipsychotics. This result of our primary analysis was confirmed in between-individual analyses, indicating the robustness of the finding. In addition, in the first-episode cohort, aHR was 0.05 (0.01–0.16) for 1.1–<1.4 DDD/day, and 0.07 (0.03–0.19) for 1.4–<1.6 DDD/day for olanzapine LAI. This finding suggests that olanzapine LAI used at these doses is relatively more effective in reducing rehospitalizations than other antipsychotics and, even more pronounced, among first-episode patients. Moreover, the only randomized controlled dose-finding study of olanzapine LAI also suggested that at the highest dose examined, 300 mg biweekly, a plateau of the dose-response curve had not been reached yet.^6,24^ However, the superior effectiveness of olanzapine LAI for relapse prevention must be balanced with the well-known adverse effects of this medication, including postinjection syndrome and metabolic adverse effects.^25–28^ Moreover, as indicated before,^22, 29–31^ overall, LAIs were among the most effective treatments, and among oral compounds clozapine and olanzapine were most effective in standard doses. Antipsychotic polypharmacy is rather common and has recently become more acceptable option^32^ but, due to enormous complexity of DDD analyses on a large number of specific antipsychotic combinations, we did not investigate this issue.

Commonly used antipsychotics were used with very different dose ranges in this real-world cohort of patients with schizophrenia. Oral olanzapine was commonly used in high dose (>1.6 DDDs/day), whereas risperidone and quetiapine were used in low dose (<0.6 DDDs/day). Many antipsychotics were relatively rarely used as 1 DDD/day (corresponding to 0.9–1.1 category in our study). This was found for most commonly used oral antipsychotics olanzapine which was more often used with higher dose, and for risperidone and quetiapine which were used with low dose more often than with standard dose. This demonstrates that general assumption of 1 DDD/day use applied in some previous studies is not a valid assumption for all patients in modeling antipsychotic use from register-based data, and that modeling has to be based on more sophisticated methods such as PRE2DUP. As DDDs have been developed as a tool for drug utilization research, for example, to monitor drug consumption over time, there is a reluctance to chance them.^33^ However, in extreme cases such as perphenazine, they should be changed. It is important to note that our estimates on the used doses are not based on what was prescribed but on what the patients actually had available them based on what and when they picked up the antipsychotic medications from pharmacies.

Results of this study need to be interpreted within its limitations. First, our results are based on a Finnish nationwide cohort including all patients hospitalized with schizophrenia diagnosis. Thus, generalizability is not an issue concerning Finland – and probably also other higher-income countries with similar healthcare system – but the results may not apply to middle- and low-income countries without full reimbursement of medication costs for patients with schizophrenia. Second, our analyses were based on antipsychotic prescriptions dispensed from pharmacies, and it was not possible to assess how much of the dispensed medication had been actually used. However, blood level analyses have shown that our drug use modeling method determines the actual medication use rather accurately.^11^ Third, the DDD methodology simply derives the DDDs of LAIs from the average recommended oral doses divided by the dosing interval, which may not always be appropriate as it has been shown eg for paliperidone, which was not included here.^34^ Moreover, current DDDs may be mainly suitable for average persons, ie, weighting 70 kg. Fourth, we only focused on the 15 most commonly used antipsychotics in Finland. Therefore, data are lacking in this study on other antipsychotics that may be of interest. Amisulpride was not included in the analysis because it does not have marketing approval in Finland. Fifth, since observational studies are prone to selection bias, we used within-individual analysis to eliminate selection biases related to patient characteristics, such as sex, genetics, and initial severity of the illness. The duration of the illness, the temporal order of treatments, as well as concomitant use of antidepressants and benzodiazepines were adjusted for. However, we cannot exclude residual confounding in this nonrandomized study design. Because within-individual analysis includes only those patients with an outcome and variation in the exposure (not the same treatment all of the time), we also conducted between-individual analysis in which all patients were included. The results of those two complementary analyses were highly consistent with each other, strengthening our findings. Sixth, since our database does not include data on the reasons for drug discontinuation, it was not possible to include those in the analyses. Seventh, despite clinical relevance of antipsychotic polypharmacy, the highly complex analyses of DDD on a large number of specific antipsychotic combinations were beyond the scope of the study. Future analyses should focus on this issue specifically. Nevertheless, despite these limitations, our analyses provide relevant information on the clinically most effective doses of most antipsychotics for the maintenance treatment of schizophrenia occurring in real-world treatment settings.

## Conclusions

Our results indicate that generally, the dose-response curves of antipsychotics followed a J- or U-shaped curve and relapse risk increased especially at low doses <0.6 DDDs/day or above ≥1.6 DDDs/day. Exceptions to this general rule were perphenazine and risperidone, with results indicating that the current standard doses by WHO are clearly too high for perphenazine and somewhat too high for risperidone for optimal maintenance treatment in relapse prevention. Olanzapine LAI at relatively high doses appeared to have markedly higher effectiveness in relapse prevention than any dose of any other antipsychotic. Finally, overall, LAIs had superior effectiveness for relapse prevention in this nationwide database study than oral antipsychotic formulations, excluding clozapine.

## Supplementary Material

sbac039_suppl_Supplementary_Material

## Data Availability

Data collected for this study is proprietary of the Finnish government agencies Social Insurance Institution of Finland and National Institute for Health and Welfare which granted researchers permission and access to data. The data that support findings of this study are available from these authorities, but restrictions apply to the availability of these data. The code used to analyze these data is available upon request by the corresponding author for purposes of reproducing the results.
