# Correction to: Optimal Doses of Specific Antipsychotics for Relapse Prevention in a Nationwide Cohort of Patients with Schizophrenia

**DOI:** 10.1093/schbul/sbac104

**Published:** 2022-08-10

This is a correction to: Heidi Taipale, Antti Tanskanen, Jurjen J Luykx, Marco Solmi, Stefan Leucht, Christoph U Correll, Jari Tiihonen, Optimal Doses of Specific Antipsychotics for Relapse Prevention in a Nationwide Cohort of Patients with Schizophrenia, Schizophrenia Bulletin, Volume 48, Issue 4, July 2022, Pages 774–784, https://doi.org/10.1093/schbul/sbac039

In the originally published version of this manuscript, three incorrect P-values were given in Table 1:

**Table:** 

Drug	Dose	aHR (95% CI)	P-value	Users	PYs	Events
Haloperidol	0.6 – <0.9	0.66 (0.57 – 0.77)	** 0.0827 **	829	767	292
Quetiapine	0.6 – <0.9	0.79 (0.73 – 0.86)	** .828 **	3867	3560	1040
Quetiapine	<0.6	0.79 (0.75 – 0.84)	** .9391 **	7633	12 081	2510

In all three instances, the correct P-value is <.0001.

Furthermore, erroneous text ‘FPO’ was included in Figure 1.

These errors have been corrected online.

