# Supplementary material for: Optimal Doses of Specific Antipsychotics for Relapse Prevention in a Nationwide Cohort of Patients with Schizophrenia
Source: Schizophr Bull. 2022 May 7;48(4):774–84. doi: 10.1093/schbul/sbac039 (PMC9212108; doi:10.1093/schbul/sbac039)
Supplement: sbac039_suppl_Supplementary_Material [file sbac039_suppl_supplementary_material.docx]

**Supplementary Material**

**Optimal Doses of Specific Antipsychotics for Relapse Prevention in a Nation-Wide Cohort of Patients with Schizophrenia**

**Supplementary Table 1.** Defined daily doses (DDDs) determined by World Health Organization (https://www.whocc.no/ddd/definition_and_general_considera/)

|  | DDD reference value as  mg/day |
| --- | --- |
| **Second generation orals** |  |
| Aripiprazole oral | 15.0 |
| Clozapine oral | 300 |
| Olanzapine oral | 10.0 |
| Quetiapine oral | 400 |
| Risperidone oral | 5.0 |
| Sertindole oral | 16.0 |
| Ziprasidone oral | 80.0 |
| **Second generation LAIs** |  |
| Olanzapine LAI | 10.0 |
| Paliperidone LAI | 2.5 |
| Risperidone LAI | 2.7 |
| **First generation orals** |  |
| Chlorprothixene | 300 |
| Flupentixol oral | 6.0 |
| Haloperidol oral | 8.0 |
| Levomepromazine oral | 300 |
| Melperone | 300 |
| Perphenazine oral | 30.0 |
| Zuclopenthixol oral | 30.0 |
| **First generation LAIs** |  |
| Fluphenazine LAI | 1.0 |
| Flupentixol LAI | 4.0 |
| Haloperidol LAI | 3.3 |
| Perphenazine LAI | 7.0 |
| Zuclopenthixol LAI | 15.0 |

Paliperidone LAI refers to once-monthly injection.

| **Supplementary Table 2.** The risk of relapse associated with specific dose categories (in DDDs per day) of specific antipsychotic monotherapies when the first 30 days were censored from all exposure periods, compared with non-use of antipsychotics in within-individual design. DDD: Defined Daily Dose. LAI=long-acting injectable antipsychotic. aHR: adjusted Hazard Ratio | | | | | | |
| --- | --- | --- | --- | --- | --- | --- |
| **Drug** | **Dose in DDDs** | **aHR (95% CI)** | **p-value** | **Users** | **PYs** | **Events** |
| Levomepromazine | <0.6 | 0.74 (0.68-0.80) | <.0001 | 3787 | 7879 | 1123 |
|  | 0.6-<0.9 | 0.61 (0.49-0.76) | <.0001 | 607 | 749 | 125 |
|  | 0.9-<1.1 | 0.51 (0.34-0.76) | 0.0012 | 307 | 272 | 39 |
|  | 1.1-<1.4 | 0.68 (0.44-1.04) | 0.0764 | 241 | 215 | 40 |
|  | 1.4-<1.6 | 0.52 (0.24-1.13) | 0.0989 | 107 | 57 | 10 |
|  | ≥1.6 | 0.71 (0.48-1.04) | 0.08 | 155 | 136 | 46 |
| Perphenazine | <0.6 | 0.77 (0.73-0.82) | <.0001 | 5900 | 20668 | 2495 |
|  | 0.6-<0.9 | 0.84 (0.73-0.96) | 0.009 | 1529 | 1991 | 396 |
|  | 0.9-<1.1 | 1.41 (1.09-1.83) | 0.009 | 529 | 254 | 109 |
|  | 1.1-<1.4 | 1.92 (1.24-2.96) | 0.0032 | 280 | 90 | 43 |
|  | 1.4-<1.6 | 1.31 (0.55-3.09) | 0.5432 | 102 | 11 | 9 |
|  | ≥1.6 | 2.28 (0.97-5.36) | 0.0588 | 97 | 10 | 14 |
| Perphenazine LAI | <0.6 | 0.46 (0.40-0.51) | <.0001 | 1415 | 3563 | 441 |
|  | 0.6-<0.9 | 0.46 (0.41-0.51) | <.0001 | 1780 | 2970 | 487 |
|  | 0.9-<1.1 | 0.54 (0.46-0.62) | <.0001 | 1237 | 1496 | 282 |
|  | 1.1-<1.4 | 0.52 (0.45-0.60) | <.0001 | 1308 | 1154 | 273 |
|  | 1.4-<1.6 | 0.59 (0.46-0.76) | <.0001 | 609 | 300 | 82 |
|  | ≥1.6 | 0.67 (0.59-0.77) | <.0001 | 833 | 1076 | 373 |
| Haloperidol | <0.6 | 0.66 (0.60-0.73) | <.0001 | 2393 | 6065 | 758 |
|  | 0.6-<0.9 | 0.66 (0.55-0.79) | <.0001 | 689 | 645 | 192 |
|  | 0.9-<1.1 | 0.67 (0.53-0.84) | 0.0005 | 465 | 494 | 126 |
|  | 1.1-<1.4 | 0.79 (0.62-1.00) | 0.0454 | 408 | 262 | 113 |
|  | 1.4-<1.6 | 0.84 (0.60-1.19) | 0.3236 | 249 | 186 | 52 |
|  | ≥1.6 | 0.99 (0.81-1.21) | 0.9277 | 361 | 422 | 173 |
| Haloperidol LAI | <0.6 | 0.50 (0.41-0.62) | <.0001 | 564 | 1575 | 148 |
|  | 0.6-<0.9 | 0.55 (0.45-0.67) | <.0001 | 720 | 1038 | 167 |
|  | 0.9-<1.1 | 0.43 (0.35-0.53) | <.0001 | 712 | 1060 | 159 |
|  | 1.1-<1.4 | 0.59 (0.49-0.70) | <.0001 | 719 | 739 | 187 |
|  | 1.4-<1.6 | 0.48 (0.37-0.61) | <.0001 | 499 | 384 | 90 |
|  | ≥1.6 | 0.70 (0.63-0.77) | <.0001 | 868 | 1624 | 662 |
| Chlorprothixene | <0.6 | 0.72 (0.65-0.79) | <.0001 | 3313 | 9572 | 960 |
|  | 0.6-<0.9 | 0.61 (0.52-0.72) | <.0001 | 1329 | 2270 | 238 |
|  | 0.9-<1.1 | 0.65 (0.52-0.81) | <.0001 | 793 | 953 | 136 |
|  | 1.1-<1.4 | 0.65 (0.51-0.83) | 0.0006 | 681 | 695 | 104 |
|  | 1.4-<1.6 | 0.83 (0.58-1.21) | 0.3336 | 359 | 186 | 44 |
|  | ≥1.6 | 0.84 (0.68-1.04) | 0.1012 | 483 | 503 | 163 |
| Zuclopenthixol | <0.6 | 0.74 (0.63-0.86) | 0.0001 | 938 | 2149 | 295 |
|  | 0.6-<0.9 | 0.46 (0.37-0.58) | <.0001 | 590 | 970 | 110 |
|  | 0.9-<1.1 | 0.52 (0.37-0.73) | 0.0002 | 364 | 364 | 53 |
|  | 1.1-<1.4 | 0.57 (0.41-0.80) | 0.001 | 334 | 249 | 63 |
|  | 1.4-<1.6 | 0.53 (0.32-0.90) | 0.0189 | 196 | 112 | 23 |
|  | ≥1.6 | 0.60 (0.49-0.74) | <.0001 | 421 | 872 | 189 |
| Zuclopenthixol LAI | <0.6 | 0.45 (0.39-0.51) | <.0001 | 1412 | 3961 | 412 |
|  | 0.6-<0.9 | 0.47 (0.42-0.52) | <.0001 | 1853 | 3471 | 567 |
|  | 0.9-<1.1 | 0.50 (0.44-0.56) | <.0001 | 1697 | 2638 | 430 |
|  | 1.1-<1.4 | 0.58 (0.52-0.66) | <.0001 | 1366 | 1275 | 400 |
|  | 1.4-<1.6 | 0.61 (0.52-0.72) | <.0001 | 872 | 487 | 203 |
|  | ≥1.6 | 0.62 (0.57-0.67) | <.0001 | 1491 | 2566 | 1087 |
| Clozapine | <0.6 | 0.55 (0.51-0.58) | <.0001 | 5148 | 8760 | 1723 |
|  | 0.6-<0.9 | 0.46 (0.43-0.49) | <.0001 | 7289 | 12369 | 2127 |
|  | 0.9-<1.1 | 0.43 (0.40-0.46) | <.0001 | 7349 | 9673 | 1525 |
|  | 1.1-<1.4 | 0.46 (0.43-0.49) | <.0001 | 7472 | 12356 | 2195 |
|  | 1.4-<1.6 | 0.62 (0.57-0.67) | <.0001 | 5525 | 3526 | 1041 |
|  | ≥1.6 | 0.54 (0.51-0.57) | <.0001 | 5568 | 14715 | 3807 |
| Olanzapine | <0.6 | 0.54 (0.50-0.59) | <.0001 | 5534 | 9010 | 1110 |
|  | 0.6-<0.9 | 0.56 (0.52-0.60) | <.0001 | 6789 | 8311 | 1232 |
|  | 0.9-<1.1 | 0.40 (0.37-0.43) | <.0001 | 6599 | 10793 | 1013 |
|  | 1.1-<1.4 | 0.67 (0.62-0.72) | <.0001 | 6730 | 4497 | 1076 |
|  | 1.4-<1.6 | 0.47 (0.43-0.51) | <.0001 | 5345 | 5349 | 782 |
|  | ≥1.6 | 0.58 (0.56-0.61) | <.0001 | 9635 | 24008 | 5336 |
| Olanzapine LAI | <0.6 | 0.81 (0.24-2.75) | 0.7401 | 25 | 9 | 6 |
|  | 0.6-<0.9 | 0.68 (0.40-1.17) | 0.1624 | 120 | 68 | 21 |
|  | 0.9-<1.1 | 0.23 (0.12-0.44) | <.0001 | 188 | 118 | 13 |
|  | 1.1-<1.4 | 0.16 (0.09-0.28) | <.0001 | 310 | 161 | 16 |
|  | 1.4-<1.6 | 0.19 (0.12-0.30) | <.0001 | 327 | 217 | 28 |
|  | ≥1.6 | 0.38 (0.32-0.45) | <.0001 | 645 | 717 | 233 |
| Quetiapine | <0.6 | 0.83 (0.77-0.89) | <.0001 | 6362 | 10776 | 1760 |
|  | 0.6-<0.9 | 0.82 (0.74-0.90) | <.0001 | 3291 | 2955 | 692 |
|  | 0.9-<1.1 | 0.73 (0.64-0.82) | <.0001 | 2380 | 2154 | 470 |
|  | 1.1-<1.4 | 0.75 (0.67-0.85) | <.0001 | 2598 | 1634 | 471 |
|  | 1.4-<1.6 | 0.71 (0.62-0.82) | <.0001 | 1998 | 1665 | 347 |
|  | ≥1.6 | 0.94 (0.87-1.01) | 0.0792 | 3054 | 3633 | 1508 |
| Risperidone | <0.6 | 0.66 (0.62-0.70) | <.0001 | 8397 | 20441 | 2790 |
|  | 0.6-<0.9 | 0.55 (0.51-0.59) | <.0001 | 5114 | 8165 | 1190 |
|  | 0.9-<1.1 | 0.71 (0.63-0.81) | <.0001 | 2557 | 1420 | 420 |
|  | 1.1-<1.4 | 0.58 (0.52-0.65) | <.0001 | 2341 | 2638 | 484 |
|  | 1.4-<1.6 | 0.86 (0.69-1.06) | 0.1575 | 992 | 442 | 140 |
|  | ≥1.6 | 1.07 (0.93-1.25) | 0.3431 | 1277 | 709 | 344 |
| Risperidone LAI | <0.6 | 0.51 (0.42-0.63) | <.0001 | 934 | 687 | 138 |
|  | 0.6-<0.9 | 0.46 (0.41-0.51) | <.0001 | 2093 | 2980 | 473 |
|  | 0.9-<1.1 | 0.36 (0.32-0.42) | <.0001 | 1731 | 2375 | 289 |
|  | 1.1-<1.4 | 0.41 (0.36-0.46) | <.0001 | 1692 | 2330 | 411 |
|  | 1.4-<1.6 | 0.98 (0.80-1.21) | 0.8635 | 823 | 241 | 149 |
|  | ≥1.6 | 1.11 (0.92-1.34) | 0.2941 | 709 | 249 | 183 |
| Aripiprazole | <0.6 | 0.53 (0.42-0.68) | <.0001 | 747 | 702 | 120 |
|  | 0.6-<0.9 | 0.58 (0.48-0.71) | <.0001 | 1114 | 881 | 163 |
|  | 0.9-<1.1 | 0.46 (0.37-0.56) | <.0001 | 1266 | 1538 | 159 |
|  | 1.1-<1.4 | 0.93 (0.73-1.19) | 0.581 | 958 | 398 | 125 |
|  | 1.4-<1.6 | 0.62 (0.44-0.87) | 0.0062 | 480 | 194 | 51 |
|  | ≥1.6 | 0.66 (0.56-0.77) | <.0001 | 1012 | 1171 | 297 |

**Supplementary Table 3.** The risk of relapse associated with specific dose categories (in DDDs per day) of specific antipsychotic monotherapies when compared with the standard dose (0.9-<1.1 DDDs/day) of the same drug in within-individual design. DDD: Defined Daily Dose. LAI=long-acting injectable antipsychotic. aHR: adjusted Hazard Ratio.

**Drug Dose in DDDs aHR (95%CI)**

Levomepromazine <0.6 1.18 (0.87-1.60)

0.6-<0.9 1.06 (0.75-1.49)

1.1-<1.4 1.20 (0.79-1.83)

1.4-<1.6 1.74 (1.01-2.99)

≥1.6 1.58 (1.06-2.37)

Perphenazine <0.6 0.54 (0.44-0.66)

0.6-<0.9 0.63 (0.50-0.78)

1.1-<1.4 1.19 (0.87-1.63)

1.4-<1.6 1.92 (1.25-2.96)

≥1.6 2.53 (1.72-3.74)

Perphenazine LAI <0.6 0.93 (0.80-1.09)

0.6-<0.9 0.89 (0.77-1.04)

1.1-<1.4 0.98 (0.83-1.16)

1.4-<1.6 1.19 (0.95-1.49)

≥1.6 1.51 (1.28-1.77)

Haloperidol <0.6 1.07 (0.88-1.30)

0.6-<0.9 1.02 (0.81-1.28)

1.1-<1.4 1.05 (0.81-1.36)

1.4-<1.6 1.18 (0.85-1.65)

≥1.6 1.66 (1.32-2.10)

Haloperidol LAI <0.6 1.07 (0.84-1.37)

0.6-<0.9 1.15 (0.92-1.45)

1.1-<1.4 1.30 (1.06-1.61)

1.4-<1.6 1.00 (0.77-1.30)

≥1.6 1.44 (1.20-1.72)

Chlorprothixene <0.6 1.01 (0.84-1.20)

0.6-<0.9 0.83 (0.68-1.03)

1.1-<1.4 1.04 (0.82-1.31)

1.4-<1.6 1.09 (0.80-1.48)

≥1.6 1.42 (1.14-1.77)

Zuclopenthixol <0.6 1.46 (1.08-1.98)

0.6-<0.9 0.99 (0.72-1.38)

1.1-<1.4 1.39 (0.96-2.00)

1.4-<1.6 1.57 (1.00-2.45)

≥1.6 1.42 (1.03-1.96)

Zuclopenthixol LAI <0.6 0.88 (0.76-1.02)

0.6-<0.9 0.90 (0.79-1.02)

1.1-<1.4 1.18 (1.03-1.35)

1.4-<1.6 1.13 (0.96-1.34)

≥1.6 1.33 (1.18-1.49)

Clozapine <0.6 1.33 (1.25-1.43)

0.6-<0.9 1.10 (1.04-1.18)

1.1-<1.4 1.04 (0.98-1.10)

1.4-<1.6 1.23 (1.15-1.32)

≥1.6 1.40 (1.32-1.49)

Olanzapine <0.6 1.32 (1.20-1.44)

0.6-<0.9 1.30 (1.20-1.42)

1.1-<1.4 1.42 (1.30-1.55)

1.4-<1.6 1.11 (1.01-1.22)

≥1.6 1.45 (1.35-1.56)

Olanzapine LAI <0.6 4.52 (1.43-14.32)

0.6-<0.9 3.18 (1.61-6.28)

1.1-<1.4 1.15 (0.61-2.17)

1.4-<1.6 0.80 (0.42-1.53)

≥1.6 1.72 (1.01-2.94)

Quetiapine <0.6 1.12 (1.00-1.25)

0.6-<0.9 1.11 (0.98-1.26)

1.1-<1.4 1.03 (0.90-1.18)

1.4-<1.6 1.05 (0.91-1.21)

≥1.6 1.40 (1.25-1.56)

Risperidone <0.6 0.94 (0.85-1.04)

0.6-<0.9 0.80 (0.72-0.89)

1.1-<1.4 0.97 (0.86-1.10)

1.4-<1.6 1.38 (1.16-1.65)

≥1.6 1.98 (1.74-2.25)

Risperidone LAI <0.6 1.24 (1.01-1.53)

0.6-<0.9 1.22 (1.05-1.41)

1.1-<1.4 1.21 (1.04-1.40)

1.4-<1.6 2.11 (1.73-2.58)

≥1.6 3.86 (3.28-4.56)

Aripiprazole <0.6 1.04 (0.80-1.36)

0.6-<0.9 1.09 (0.87-1.37)

1.1-<1.4 1.71 (1.34-2.17)

1.4-<1.6 1.33 (0.97-1.82)

≥1.6 1.53 (1.26-1.88)

**Supplementary Table 4**. The risk of relapse associated with specific dose categories (in DDDs per day) of specific antipsychotic monotherapies when compared with most commonly used antipsychotic/ dose combination, which was oral olanzapine >1.6 DDDs/day, in within-individual design. DDD: Defined Daily Dose. LAI=long-acting injectable antipsychotic. aHR: adjusted Hazard Ratio.

**Drug Dose in DDDs aHR (95% CI)**

Olanzapine LAI 1.4-<1.6 0.29 (0.20-0.43)

Olanzapine LAI 0.9-<1.1 0.36 (0.22-0.61)

Olanzapine LAI 1.1-<1.4 0.42 (0.28-0.61)

Olanzapine LAI ≥1.6 0.62 (0.54-0.72)

Risperidone LAI 0.9-<1.1 0.63 (0.56-0.71)

Clozapine 0.9-<1.1 0.64 (0.61-0.68)

Clozapine 1.1-<1.4 0.67 (0.63-0.70)

Olanzapine 0.9-<1.1 0.69 (0.64-0.74)

Clozapine 0.6-<0.9 0.71 (0.67-0.75)

Zuclopenthixol LAI <0.6 0.71 (0.63-0.80)

Perphenazine LAI 0.6-<0.9 0.72 (0.65-0.80)

Zuclopenthixol LAI 0.6-<0.9 0.73 (0.66-0.80)

Perphenazine LAI <0.6 0.75 (0.67-0.84)

Risperidone LAI 1.1-<1.4 0.76 (0.68-0.84)

Risperidone LAI 0.6-<0.9 0.76 (0.69-0.84)

Haloperidol LAI 0.9-<1.1 0.76 (0.65-0.90)

Olanzapine 1.4-<1.6 0.76 (0.71-0.82)

Haloperidol LAI 1.4-<1.6 0.77 (0.62-0.95)

Risperidone LAI <0.6 0.78 (0.65-0.93)

Perphenazine LAI 1.1-<1.4 0.79 (0.69-0.89)

Zuclopenthixol 0.6-<0.9 0.79 (0.65-0.95)

Zuclopenthixol 0.9-<1.1 0.79 (0.60-1.05)

Clozapine 1.4-<1.6 0.79 (0.75-0.84)

Perphenazine LAI 0.9-<1.1 0.80 (0.71-0.91)

Zuclopenthixol LAI 0.9-<1.1 0.81 (0.73-0.90)

Haloperidol LAI <0.6 0.82 (0.68-0.99)

Clozapine <0.6 0.86 (0.81-0.91)

Aripiprazole 0.9-<1.1 0.88 (0.75-1.03)

Haloperidol LAI 0.6-<0.9 0.88 (0.74-1.04)

Olanzapine 0.6-<0.9 0.90 (0.84-0.96)

Clozapine ≥1.6 0.90 (0.86-0.95)

Olanzapine <0.6 0.91 (0.84-0.98)

Aripiprazole <0.6 0.91 (0.74-1.13)

Zuclopenthixol LAI 1.4-<1.6 0.92 (0.80-1.06)

Risperidone 0.6-<0.9 0.93 (0.87-0.99)

Perphenazine LAI 1.4-<1.6 0.96 (0.78-1.16)

Aripiprazole 0.6-<0.9 0.96 (0.81-1.14)

Zuclopenthixol LAI 1.1-<1.4 0.96 (0.86-1.07)

Olanzapine 1.1-<1.4 0.98 (0.92-1.05)

Haloperidol LAI 1.1-<1.4 0.99 (0.85-1.16)

Olanzapine ≥1.6 REFERENCE

Chlorprotixene 0.6-<0.9 1.04 (0.90-1.19)

Zuclopenthixol LAI ≥1.6 1.08 (1.00-1.16)

Risperidone <0.6 1.09 (1.03-1.15)

Haloperidol LAI ≥1.6 1.10 (1.00-1.21)

Levomepromazine 0.9-<1.1 1.10 (0.81-1.48)

Zuclopenthixol 1.1-<1.4 1.10 (0.85-1.41)

Haloperidol 0.9-<1.1 1.11 (0.92-1.34)

Zuclopenthixol ≥1.6 1.12 (0.95-1.33)

Risperidone 1.1-<1.4 1.12 (1.03-1.23)

Haloperidol 0.6-<0.9 1.13 (0.98-1.32)

Olanzapine LAI 0.6-<0.9 1.15 (0.73-1.81)

Zuclopenthixol <0.6 1.16 (1.01-1.32)

Risperidone 0.9-<1.1 1.16 (1.05-1.28)

Levomepromazine 0.6-<0.9 1.16 (0.97-1.39)

Haloperidol 1.1-<1.4 1.16 (0.96-1.42)

Aripiprazole 1.4-<1.6 1.17 (0.89-1.53)

Haloperidol <0.6 1.19 (1.08-1.30)

Perphenazine LAI ≥1.6 1.21 (1.08-1.36)

Quetiapine 0.9-<1.1 1.22 (1.10-1.36)

Perphenazine <0.6 1.23 (1.16-1.31)

Zuclopenthixol 1.4-<1.6 1.24 (0.86-1.78)

Chlorprotixene 0.9-<1.1 1.24 (1.05-1.47)

Chlorprotixene <0.6 1.25 (1.16-1.36)

Quetiapine 1.1-<1.4 1.26 (1.14-1.39)

Quetiapine 1.4-<1.6 1.28 (1.14-1.43)

Chlorprotixene 1.1-<1.4 1.29 (1.08-1.54)

Levomepromazine <0.6 1.29 (1.20-1.39)

Haloperidol 1.4-<1.6 1.31 (0.99-1.74)

Levomepromazine 1.1-<1.4 1.32 (0.96-1.81)

Risperidone LAI 1.4-<1.6 1.32 (1.12-1.57)

Aripiprazole ≥1.6 1.35 (1.18-1.53)

Quetiapine 0.6-<0.9 1.35 (1.24-1.47)

Chlorprotixene 1.4-<1.6 1.35 (1.04-1.76)

Quetiapine <0.6 1.36 (1.27-1.45)

Perphenazine 0.6-<0.9 1.42 (1.27-1.59)

Aripiprazole 1.1-<1.4 1.50 (1.24-1.80)

Risperidone 1.4-<1.6 1.60 (1.38-1.87)

Olanzapine LAI <0.6 1.64 (0.58-4.60)

Quetiapine ≥1.6 1.71 (1.60-1.82)

Levomepromazine ≥1.6 1.74 (1.32-2.28)

Chlorprotixene ≥1.6 1.77 (1.51-2.07)

Haloperidol ≥1.6 1.84 (1.58-2.15)

Levomepromazine 1.4-<1.6 1.91 (1.21-3.01)

Perphenazine 0.9-<1.1 2.27 (1.86-2.77)

Risperidone ≥1.6 2.30 (2.08-2.54)

Risperidone LAI ≥1.6 2.42 (2.13-2.74)

Perphenazine 1.1-<1.4 2.70 (2.08-3.51)

Perphenazine 1.4-<1.6 4.36 (2.92-6.51)

Perphenazine ≥1.6 5.75 (4.06-8.14)

| **Supplementary Table 5.** The risk of relapse associated with use of specific antipsychotic monotherapies with specific dose (in DDDs per day) in traditional between-individual model. The reference is oral olanzapine >1.6 DDDs/day. DDD: Defined Daily Dose. LAI: long-acting injectable antipsychotic. HR: adjusted Hazard Ratio. | | |
| --- | --- | --- |
| **Drug** | **Dose in DDDs** | **HR (95% CI)** |
| Olanzapine LAI | 1.4-<1.6 | 0.37 (0.21-0.66) |
| Olanzapine LAI | 0.9-<1.1 | 0.45 (0.27-0.75) |
| Haloperidol LAI | <0.6 | 0.46 (0.37-0.58) |
| Zuclopenthixol LAI | <0.6 | 0.47 (0.40-0.55) |
| Olanzapine LAI | 1.1-<1.4 | 0.49 (0.34-0.71) |
| Olanzapine | 0.9-<1.1 | 0.55 (0.50-0.60) |
| Risperidone LAI | 0.9-<1.1 | 0.58 (0.50-0.67) |
| Perphenazine LAI | <0.6 | 0.58 (0.50-0.69) |
| Zuclopenthixol LAI | 0.6-<0.9 | 0.62 (0.54-0.71) |
| Chlorprotixene | 0.6-<0.9 | 0.62 (0.53-0.73) |
| Aripiprazole | 0.9-<1.1 | 0.62 (0.52-0.74) |
| Zuclopenthixol | 0.6-<0.9 | 0.63 (0.50-0.79) |
| Clozapine | 0.9-<1.1 | 0.64 (0.59-0.70) |
| Haloperidol LAI | 0.9-<1.1 | 0.65 (0.50-0.85) |
| Haloperidol LAI | 0.6-<0.9 | 0.66 (0.54-0.81) |
| Zuclopenthixol | 0.9-<1.1 | 0.67 (0.49-0.92) |
| Clozapine | 1.1-<1.4 | 0.68 (0.63-0.73) |
| Zuclopenthixol LAI | 0.9-<1.1 | 0.69 (0.60-0.80) |
| Perphenazine LAI | 0.6-<0.9 | 0.70 (0.59-0.82) |
| Zuclopenthixol | <0.6 | 0.70 (0.58-0.85) |
| Chlorprotixene | <0.6 | 0.71 (0.64-0.79) |
| Olanzapine | 1.4-<1.6 | 0.71 (0.65-0.79) |
| Olanzapine | <0.6 | 0.72 (0.66-0.79) |
| Clozapine | 0.6-<0.9 | 0.73 (0.68-0.80) |
| Haloperidol | <0.6 | 0.77 (0.68-0.87) |
| Perphenazine LAI | 0.9-<1.1 | 0.77 (0.63-0.96) |
| Haloperidol LAI | 1.4-<1.6 | 0.78 (0.56-1.08) |
| Olanzapine | 0.6-<0.9 | 0.78 (0.72-0.85) |
| Risperidone | 0.6-<0.9 | 0.79 (0.72-0.87) |
| Risperidone LAI | 0.6-<0.9 | 0.80 (0.68-0.93) |
| Perphenazine | <0.6 | 0.81 (0.75-0.88) |
| Levomepromazine | 0.9-<1.1 | 0.81 (0.56-1.17) |
| Risperidone LAI | 1.1-<1.4 | 0.82 (0.69-0.97) |
| Aripiprazole | 0.6-<0.9 | 0.82 (0.68-1.00) |
| Haloperidol LAI | 1.1-<1.4 | 0.85 (0.69-1.05) |
| Aripiprazole | <0.6 | 0.85 (0.69-1.05) |
| Risperidone | <0.6 | 0.87 (0.81-0.94) |
| Chlorprotixene | 0.9-<1.1 | 0.88 (0.72-1.08) |
| Perphenazine LAI | 1.1-<1.4 | 0.88 (0.74-1.05) |
| Chlorprotixene | 1.1-<1.4 | 0.88 (0.71-1.10) |
| Clozapine | <0.6 | 0.89 (0.81-0.97) |
| Levomepromazine | <0.6 | 0.90 (0.82-0.99) |
| Clozapine | 1.4-<1.6 | 0.90 (0.82-0.98) |
| Risperidone LAI | <0.6 | 0.90 (0.76-1.07) |
| Zuclopenthixol | ≥1.6 | 0.93 (0.72-1.18) |
| Clozapine | ≥1.6 | 0.93 (0.86-1.00) |
| Zuclopenthixol | 1.4-<1.6 | 0.96 (0.65-1.42) |
| Levomepromazine | 1.1-<1.4 | 0.96 (0.68-1.36) |
| Risperidone | 1.1-<1.4 | 0.97 (0.85-1.11) |
| Olanzapine | ≥1.6 | 1.00 (-) |
| Olanzapine | 1.1-<1.4 | 1.01 (0.93-1.09) |
| Levomepromazine | 0.6-<0.9 | 1.01 (0.77-1.33) |
| Olanzapine LAI | ≥1.6 | 1.05 (0.76-1.44) |
| Olanzapine LAI | 0.6-<0.9 | 1.07 (0.52-2.20) |
| Zuclopenthixol LAI | 1.1-<1.4 | 1.07 (0.91-1.26) |
| Haloperidol | 1.4-<1.6 | 1.07 (0.74-1.57) |
| Aripiprazole | 1.4-<1.6 | 1.08 (0.80-1.46) |
| Quetiapine | <0.6 | 1.08 (1.00-1.17) |
| Quetiapine | 0.9-<1.1 | 1.11 (0.98-1.27) |
| Quetiapine | 1.4-<1.6 | 1.12 (0.97-1.30) |
| Zuclopenthixol LAI | 1.4-<1.6 | 1.13 (0.95-1.34) |
| Haloperidol | 0.9-<1.1 | 1.13 (0.89-1.43) |
| Perphenazine | 0.6-<0.9 | 1.15 (0.98-1.34) |
| Perphenazine LAI | 1.4-<1.6 | 1.15 (0.90-1.48) |
| Haloperidol | 0.6-<0.9 | 1.16 (0.94-1.43) |
| Zuclopenthixol | 1.1-<1.4 | 1.16 (0.85-1.60) |
| Haloperidol LAI | ≥1.6 | 1.17 (1.01-1.35) |
| Chlorprotixene | 1.4-<1.6 | 1.20 (0.91-1.60) |
| Quetiapine | 0.6-<0.9 | 1.22 (1.10-1.36) |
| Zuclopenthixol LAI | ≥1.6 | 1.23 (1.10-1.39) |
| Quetiapine | 1.1-<1.4 | 1.31 (1.16-1.47) |
| Risperidone | 0.9-<1.1 | 1.31 (1.16-1.49) |
| Aripiprazole | 1.1-<1.4 | 1.31 (1.08-1.59) |
| Aripiprazole | ≥1.6 | 1.35 (1.15-1.59) |
| Perphenazine LAI | ≥1.6 | 1.36 (1.13-1.62) |
| Levomepromazine | 1.4-<1.6 | 1.38 (0.70-2.71) |
| Levomepromazine | ≥1.6 | 1.46 (1.01-2.10) |
| Haloperidol | 1.1-<1.4 | 1.47 (1.10-1.98) |
| Risperidone | 1.4-<1.6 | 1.56 (1.32-1.85) |
| Chlorprotixene | ≥1.6 | 1.61 (1.30-1.98) |
| Quetiapine | ≥1.6 | 1.82 (1.67-1.99) |
| Risperidone LAI | 1.4-<1.6 | 1.88 (1.57-2.26) |
| Perphenazine | 0.9-<1.1 | 2.02 (1.53-2.67) |
| Haloperidol | ≥1.6 | 2.07 (1.67-2.56) |
| Olanzapine LAI | <0.6 | 2.10 (1.23-3.60) |
| Risperidone | ≥1.6 | 2.65 (2.32-3.02) |
| Perphenazine | 1.1-<1.4 | 3.29 (2.50-4.32) |
| Risperidone LAI | ≥1.6 | 4.02 (3.46-4.68) |
| Perphenazine | 1.4-<1.6 | 5.65 (4.06-7.86) |
| Perphenazine | ≥1.6 | 7.25 (5.42-9.70) |

| Adjusted for age, gender, temporal order of treatment, previous number of psychiatric re-hospitalizations, calendar year, use of antidepressants, benzodiazepines and related drugs, anticholinergic antiparkinson drugs and statins, diagnosis of cardiovascular disease, diabetes, cancer, asthma/ COPD, substance abuse, suicide attempt, liver disease, renal disease. |
| --- |

**Supplementary Table 6.** The risk of re-hospitalization associated with use of specific antipsychotic monotherapies with specific dose (in DDDs per day) in within-individual design when stratified by baseline age. Reference: non-use of antipsychotics. DDD: Defined Daily Dose. LAI: long-acting injectable antipsychotic. HR: adjusted Hazard Ratio.

|  | **Dose in DDDs** | **≤45 years**  **HR (95% CI)** | **>45 years**  **HR (95% CI)** |
| --- | --- | --- | --- |
| **Drug** |  |  |  |
| Levomepromazine | <0.6 | 0.80 (0.74-0.87) | 0.72 (0.65-0.80) |
|  | 0.6-<0.9 | 0.69 (0.55-0.88) | 0.72 (0.56-0.93) |
|  | 0.9-<1.1 | 0.37 (0.22-0.62) | 1.04 (0.71-1.53) |
|  | 1.1-<1.4 | 0.74 (0.44-1.25) | 0.97 (0.65-1.44) |
|  | 1.4-<1.6 | 0.52 (0.16-1.65) | 1.57 (0.95-2.60) |
|  | ≥1.6 | 1.00 (0.69-1.46) | 1.16 (0.78-1.72) |
| Perphenazine | <0.6 | 0.69 (0.64-0.74) | 0.80 (0.73-0.86) |
|  | 0.6-<0.9 | 0.77 (0.67-0.89) | 0.95 (0.80-1.14) |
|  | 0.9-<1.1 | 1.21 (0.95-1.55) | 1.60 (1.15-2.23) |
|  | 1.1-<1.4 | 1.42 (1.03-1.95) | 1.96 (1.25-3.05) |
|  | 1.4-<1.6 | 2.18 (1.30-3.65) | 3.47 (1.86-6.48) |
|  | ≥1.6 | 2.71 (1.71-4.28) | 4.87 (2.84-8.34) |
| Perphenazine LAI | <0.6 | 0.43 (0.38-0.49) | 0.46 (0.39-0.55) |
|  | 0.6-<0.9 | 0.38 (0.33-0.43) | 0.50 (0.43-0.58) |
|  | 0.9-<1.1 | 0.42 (0.36-0.50) | 0.56 (0.47-0.68) |
|  | 1.1-<1.4 | 0.46 (0.39-0.55) | 0.50 (0.42-0.60) |
|  | 1.4-<1.6 | 0.50 (0.38-0.65) | 0.69 (0.52-0.93) |
|  | ≥1.6 | 0.59 (0.51-0.69) | 0.96 (0.80-1.14) |
| Haloperidol | <0.6 | 0.67 (0.59-0.75) | 0.77 (0.68-0.87) |
|  | 0.6-<0.9 | 0.69 (0.57-0.84) | 0.65 (0.51-0.81) |
|  | 0.9-<1.1 | 0.68 (0.52-0.87) | 0.68 (0.52-0.88) |
|  | 1.1-<1.4 | 0.53 (0.40-0.71) | 0.94 (0.72-1.22) |
|  | 1.4-<1.6 | 0.78 (0.55-1.09) | 0.77 (0.47-1.25) |
|  | ≥1.6 | 1.04 (0.84-1.28) | 1.24 (1.00-1.53) |
| Haloperidol LAI | <0.6 | 0.46 (0.34-0.62) | 0.54 (0.42-0.69) |
|  | 0.6-<0.9 | 0.44 (0.35-0.56) | 0.65 (0.51-0.83) |
|  | 0.9-<1.1 | 0.42 (0.34-0.52) | 0.49 (0.39-0.62) |
|  | 1.1-<1.4 | 0.51 (0.41-0.63) | 0.72 (0.58-0.90) |
|  | 1.4-<1.6 | 0.43 (0.32-0.57) | 0.51 (0.38-0.71) |
|  | ≥1.6 | 0.59 (0.53-0.67) | 0.76 (0.65-0.88) |
| Chlorprotixene | <0.6 | 0.73 (0.66-0.81) | 0.77 (0.68-0.87) |
|  | 0.6-<0.9 | 0.59 (0.50-0.71) | 0.65 (0.53-0.80) |
|  | 0.9-<1.1 | 0.63 (0.51-0.79) | 0.93 (0.73-1.19) |
|  | 1.1-<1.4 | 0.70 (0.56-0.89) | 0.87 (0.67-1.14) |
|  | 1.4-<1.6 | 0.76 (0.54-1.08) | 0.86 (0.57-1.29) |
|  | ≥1.6 | 0.90 (0.74-1.10) | 1.32 (1.04-1.68) |
| Zuclopenthixol | <0.6 | 0.61 (0.51-0.74) | 0.80 (0.66-0.98) |
|  | 0.6-<0.9 | 0.40 (0.31-0.53) | 0.56 (0.42-0.74) |
|  | 0.9-<1.1 | 0.61 (0.42-0.87) | 0.36 (0.23-0.55) |
|  | 1.1-<1.4 | 0.64 (0.47-0.88) | 0.67 (0.45-1.00) |
|  | 1.4-<1.6 | 0.78 (0.47-1.30) | 0.73 (0.44-1.21) |
|  | ≥1.6 | 0.68 (0.54-0.86) | 0.67 (0.53-0.86) |
| Zuclopenthixol LAI | <0.6 | 0.46 (0.39-0.54) | 0.40 (0.34-0.47) |
|  | 0.6-<0.9 | 0.40 (0.35-0.46) | 0.48 (0.42-0.55) |
|  | 0.9-<1.1 | 0.48 (0.42-0.55) | 0.49 (0.42-0.57) |
|  | 1.1-<1.4 | 0.55 (0.48-0.63) | 0.62 (0.53-0.71) |
|  | 1.4-<1.6 | 0.54 (0.45-0.64) | 0.56 (0.46-0.70) |
|  | ≥1.6 | 0.60 (0.55-0.66) | 0.70 (0.63-0.78) |
| Clozapine | <0.6 | 0.47 (0.44-0.50) | 0.57 (0.51-0.63) |
|  | 0.6-<0.9 | 0.37 (0.35-0.39) | 0.53 (0.48-0.58) |
|  | 0.9-<1.1 | 0.34 (0.32-0.36) | 0.46 (0.41-0.52) |
|  | 1.1-<1.4 | 0.35 (0.33-0.37) | 0.50 (0.46-0.56) |
|  | 1.4-<1.6 | 0.41 (0.38-0.43) | 0.64 (0.56-0.72) |
|  | ≥1.6 | 0.47 (0.45-0.49) | 0.70 (0.64-0.77) |
| Olanzapine | <0.6 | 0.56 (0.51-0.61) | 0.49 (0.43-0.54) |
|  | 0.6-<0.9 | 0.52 (0.48-0.56) | 0.53 (0.48-0.59) |
|  | 0.9-<1.1 | 0.42 (0.38-0.45) | 0.39 (0.35-0.43) |
|  | 1.1-<1.4 | 0.55 (0.50-0.59) | 0.62 (0.56-0.69) |
|  | 1.4-<1.6 | 0.40 (0.37-0.44) | 0.53 (0.48-0.60) |
|  | ≥1.6 | 0.54 (0.51-0.56) | 0.68 (0.64-0.73) |
| Olanzapine LAI | <0.6 | 0.35 (0.04-3.00) | 1.63 (0.44-6.01) |
|  | 0.6-<0.9 | 0.83 (0.50-1.38) | 0.25 (0.07-0.86) |
|  | 0.9-<1.1 | 0.27 (0.15-0.47) | 0.11 (0.03-0.36) |
|  | 1.1-<1.4 | 0.23 (0.14-0.38) | 0.29 (0.16-0.53) |
|  | 1.4-<1.6 | 0.14 (0.08-0.24) | 0.24 (0.14-0.42) |
|  | ≥1.6 | 0.31 (0.27-0.37) | 0.52 (0.40-0.67) |
| Quetiapine | <0.6 | 0.77 (0.71-0.83) | 0.86 (0.78-0.94) |
|  | 0.6-<0.9 | 0.81 (0.73-0.89) | 0.76 (0.66-0.87) |
|  | 0.9-<1.1 | 0.68 (0.60-0.77) | 0.79 (0.66-0.93) |
|  | 1.1-<1.4 | 0.74 (0.66-0.83) | 0.72 (0.61-0.85) |
|  | 1.4-<1.6 | 0.71 (0.63-0.82) | 0.79 (0.65-0.96) |
|  | ≥1.6 | 0.92 (0.86-0.99) | 1.16 (1.04-1.29) |
| Risperidone | <0.6 | 0.62 (0.58-0.67) | 0.68 (0.63-0.73) |
|  | 0.6-<0.9 | 0.53 (0.49-0.57) | 0.56 (0.51-0.63) |
|  | 0.9-<1.1 | 0.65 (0.58-0.72) | 0.74 (0.62-0.87) |
|  | 1.1-<1.4 | 0.58 (0.52-0.65) | 0.82 (0.71-0.95) |
|  | 1.4-<1.6 | 0.93 (0.77-1.11) | 0.91 (0.69-1.20) |
|  | ≥1.6 | 1.34 (1.19-1.51) | 1.32 (1.11-1.56) |
| Risperidone LAI | <0.6 | 0.51 (0.41-0.64) | 0.39 (0.29-0.51) |
|  | 0.6-<0.9 | 0.46 (0.41-0.52) | 0.44 (0.38-0.51) |
|  | 0.9-<1.1 | 0.37 (0.32-0.43) | 0.36 (0.30-0.44) |
|  | 1.1-<1.4 | 0.42 (0.37-0.47) | 0.50 (0.42-0.59) |
|  | 1.4-<1.6 | 0.62 (0.50-0.76) | 1.18 (0.90-1.55) |
|  | ≥1.6 | 1.20 (1.04-1.40) | 1.98 (1.59-2.46) |
| Aripiprazole | <0.6 | 0.59 (0.46-0.74) | 0.35 (0.22-0.57) |
|  | 0.6-<0.9 | 0.50 (0.41-0.61) | 0.75 (0.52-1.08) |
|  | 0.9-<1.1 | 0.50 (0.41-0.60) | 0.52 (0.37-0.71) |
|  | 1.1-<1.4 | 0.81 (0.65-1.00) | 1.02 (0.72-1.44) |
|  | 1.4-<1.6 | 0.69 (0.51-0.92) | 0.51 (0.25-1.04) |
|  | ≥1.6 | 0.74 (0.64-0.85) | 0.87 (0.66-1.15) |

**Supplementary Table 7.** The risk of re-hospitalization associated with use of specific antipsychotic monotherapies with specific dose (in DDDs per day) in within-individual design when stratified by schizophrenia vs. schizoaffective diagnosis. Reference: non-use of antipsychotics. DDD: Defined Daily Dose. LAI: long-acting injectable antipsychotic. HR: adjusted Hazard Ratio.

|  | **Dose in DDDs** | **Schizoaffective disorder**  **HR (95% CI)** | **Schizophrenia**  **HR (95% CI)** |
| --- | --- | --- | --- |
|  |  |  |  |
| Levomepromazine | <0.6 | 0.73 (0.64-0.84) | 0.77 (0.71-0.83) |
|  | 0.6-<0.9 | 0.80 (0.57-1.11) | 0.64 (0.52-0.79) |
|  | 0.9-<1.1 | 0.99 (0.52-1.87) | 0.58 (0.41-0.81) |
|  | 1.1-<1.4 | 0.60 (0.29-1.26) | 0.82 (0.57-1.16) |
|  | 1.4-<1.6 | 0.83 (0.24-2.90) | 1.18 (0.72-1.93) |
|  | ≥1.6 | 1.00 (0.55-1.81) | 1.03 (0.76-1.40) |
| Perphenazine | <0.6 | 0.75 (0.65-0.86) | 0.71 (0.67-0.76) |
|  | 0.6-<0.9 | 0.82 (0.57-1.18) | 0.82 (0.73-0.93) |
|  | 0.9-<1.1 | 2.45 (1.23-4.86) | 1.25 (1.02-1.54) |
|  | 1.1-<1.4 | 3.30 (1.39-7.86) | 1.46 (1.11-1.92) |
|  | 1.4-<1.6 | 2.14 (0.76-6.04) | 2.62 (1.70-4.05) |
|  | ≥1.6 | 2.23 (1.03-4.84) | 3.70 (2.53-5.43) |
| Perphenazine LAI | <0.6 | 0.39 (0.27-0.57) | 0.44 (0.39-0.49) |
|  | 0.6-<0.9 | 0.37 (0.26-0.53) | 0.42 (0.38-0.47) |
|  | 0.9-<1.1 | 0.48 (0.32-0.71) | 0.46 (0.41-0.53) |
|  | 1.1-<1.4 | 0.30 (0.18-0.50) | 0.47 (0.41-0.53) |
|  | 1.4-<1.6 | 0.69 (0.38-1.23) | 0.54 (0.44-0.66) |
|  | ≥1.6 | 0.97 (0.69-1.36) | 0.68 (0.60-0.76) |
| Haloperidol | <0.6 | 0.72 (0.59-0.88) | 0.68 (0.62-0.75) |
|  | 0.6-<0.9 | 0.68 (0.47-0.99) | 0.66 (0.56-0.77) |
|  | 0.9-<1.1 | 0.75 (0.48-1.18) | 0.63 (0.51-0.77) |
|  | 1.1-<1.4 | 0.62 (0.38-0.99) | 0.69 (0.56-0.86) |
|  | 1.4-<1.6 | 0.93 (0.38-2.24) | 0.75 (0.56-1.01) |
|  | ≥1.6 | 1.07 (0.66-1.72) | 1.07 (0.91-1.26) |
| Haloperidol LAI | <0.6 | 0.47 (0.29-0.78) | 0.48 (0.39-0.59) |
|  | 0.6-<0.9 | 0.47 (0.28-0.80) | 0.52 (0.43-0.62) |
|  | 0.9-<1.1 | 0.27 (0.16-0.46) | 0.47 (0.40-0.56) |
|  | 1.1-<1.4 | 0.47 (0.30-0.73) | 0.60 (0.51-0.70) |
|  | 1.4-<1.6 | 0.28 (0.14-0.57) | 0.47 (0.38-0.59) |
|  | ≥1.6 | 0.71 (0.56-0.91) | 0.63 (0.57-0.69) |
| Chlorprotixene | <0.6 | 0.73 (0.62-0.85) | 0.74 (0.67-0.80) |
|  | 0.6-<0.9 | 0.65 (0.48-0.89) | 0.59 (0.51-0.69) |
|  | 0.9-<1.1 | 1.16 (0.79-1.70) | 0.66 (0.55-0.79) |
|  | 1.1-<1.4 | 0.76 (0.48-1.21) | 0.75 (0.62-0.91) |
|  | 1.4-<1.6 | 1.25 (0.66-2.35) | 0.72 (0.54-0.96) |
|  | ≥1.6 | 1.57 (1.08-2.27) | 0.95 (0.80-1.12) |
| Zuclopenthixol | <0.6 | 0.74 (0.57-0.96) | 0.65 (0.56-0.77) |
|  | 0.6-<0.9 | 0.44 (0.29-0.66) | 0.47 (0.38-0.58) |
|  | 0.9-<1.1 | 0.58 (0.32-1.03) | 0.43 (0.32-0.60) |
|  | 1.1-<1.4 | 0.64 (0.39-1.05) | 0.65 (0.49-0.86) |
|  | 1.4-<1.6 | 0.76 (0.39-1.51) | 0.72 (0.47-1.10) |
|  | ≥1.6 | 0.89 (0.63-1.25) | 0.60 (0.49-0.73) |
| Zuclopenthixol LAI | <0.6 | 0.36 (0.25-0.51) | 0.42 (0.37-0.48) |
|  | 0.6-<0.9 | 0.40 (0.31-0.51) | 0.43 (0.39-0.47) |
|  | 0.9-<1.1 | 0.59 (0.46-0.76) | 0.45 (0.41-0.51) |
|  | 1.1-<1.4 | 0.61 (0.46-0.82) | 0.55 (0.49-0.61) |
|  | 1.4-<1.6 | 0.84 (0.59-1.20) | 0.50 (0.43-0.58) |
|  | ≥1.6 | 0.76 (0.63-0.91) | 0.61 (0.56-0.65) |
| Clozapine | <0.6 | 0.49 (0.43-0.55) | 0.50 (0.47-0.53) |
|  | 0.6-<0.9 | 0.44 (0.39-0.49) | 0.41 (0.39-0.43) |
|  | 0.9-<1.1 | 0.43 (0.38-0.49) | 0.36 (0.34-0.39) |
|  | 1.1-<1.4 | 0.43 (0.38-0.48) | 0.38 (0.36-0.40) |
|  | 1.4-<1.6 | 0.49 (0.43-0.57) | 0.46 (0.43-0.49) |
|  | ≥1.6 | 0.66 (0.60-0.73) | 0.50 (0.48-0.53) |
| Olanzapine | <0.6 | 0.48 (0.41-0.56) | 0.54 (0.50-0.58) |
|  | 0.6-<0.9 | 0.60 (0.52-0.69) | 0.51 (0.47-0.54) |
|  | 0.9-<1.1 | 0.42 (0.36-0.49) | 0.40 (0.37-0.43) |
|  | 1.1-<1.4 | 0.59 (0.51-0.68) | 0.57 (0.53-0.61) |
|  | 1.4-<1.6 | 0.42 (0.35-0.50) | 0.45 (0.42-0.49) |
|  | ≥1.6 | 0.63 (0.58-0.68) | 0.57 (0.55-0.60) |
| Olanzapine LAI | <0.6 | 0.95 (0.16-5.81) | 0.90 (0.25-3.22) |
|  | 0.6-<0.9 | 2.68 (1.09-6.54) | 0.41 (0.23-0.73) |
|  | 0.9-<1.1 | 0.10 (0.01-0.69) | 0.23 (0.13-0.39) |
|  | 1.1-<1.4 | 0.27 (0.10-0.69) | 0.24 (0.15-0.36) |
|  | 1.4-<1.6 | 0.26 (0.14-0.49) | 0.14 (0.09-0.23) |
|  | ≥1.6 | 0.43 (0.31-0.58) | 0.35 (0.30-0.41) |
| Quetiapine | <0.6 | 0.81 (0.72-0.92) | 0.79 (0.74-0.85) |
|  | 0.6-<0.9 | 0.84 (0.71-0.99) | 0.78 (0.71-0.86) |
|  | 0.9-<1.1 | 0.73 (0.60-0.89) | 0.71 (0.63-0.80) |
|  | 1.1-<1.4 | 0.67 (0.56-0.81) | 0.77 (0.69-0.86) |
|  | 1.4-<1.6 | 0.67 (0.54-0.82) | 0.79 (0.70-0.90) |
|  | ≥1.6 | 1.01 (0.90-1.14) | 1.00 (0.93-1.07) |
| Risperidone | <0.6 | 0.63 (0.56-0.71) | 0.64 (0.60-0.67) |
|  | 0.6-<0.9 | 0.57 (0.48-0.67) | 0.54 (0.50-0.57) |
|  | 0.9-<1.1 | 0.92 (0.72-1.16) | 0.64 (0.58-0.71) |
|  | 1.1-<1.4 | 0.76 (0.59-0.99) | 0.64 (0.58-0.70) |
|  | 1.4-<1.6 | 0.84 (0.51-1.38) | 0.94 (0.80-1.10) |
|  | ≥1.6 | 1.66 (1.26-2.18) | 1.30 (1.17-1.43) |
| Risperidone LAI | <0.6 | 0.26 (0.16-0.44) | 0.50 (0.41-0.60) |
|  | 0.6-<0.9 | 0.53 (0.42-0.66) | 0.43 (0.38-0.48) |
|  | 0.9-<1.1 | 0.41 (0.30-0.55) | 0.36 (0.32-0.40) |
|  | 1.1-<1.4 | 0.40 (0.30-0.54) | 0.45 (0.40-0.50) |
|  | 1.4-<1.6 | 0.80 (0.49-1.29) | 0.76 (0.64-0.91) |
|  | ≥1.6 | 1.24 (0.87-1.77) | 1.43 (1.25-1.63) |
| Aripiprazole | <0.6 | 0.64 (0.39-1.05) | 0.51 (0.41-0.65) |
|  | 0.6-<0.9 | 0.53 (0.37-0.77) | 0.57 (0.47-0.69) |
|  | 0.9-<1.1 | 0.47 (0.34-0.65) | 0.53 (0.44-0.64) |
|  | 1.1-<1.4 | 0.68 (0.45-1.01) | 0.95 (0.77-1.16) |
|  | 1.4-<1.6 | 1.17 (0.70-1.95) | 0.57 (0.41-0.79) |
|  | ≥1.6 | 0.70 (0.55-0.90) | 0.83 (0.72-0.96) |

**Supplementary Table 8.** The risk of re-hospitalization associated with use of specific antipsychotic monotherapies with specific dose (in DDDs per day) in within-individual design in persons with incident diagnosis of schizophrenia/ schizoaffective disorder at baseline. Reference: non-use of antipsychotics. DDD: Defined Daily Dose. LAI: long-acting injectable antipsychotic. HR: adjusted Hazard Ratio.

| **Drug** | **Dose in DDDs** | **HR (95% CI)** |
| --- | --- | --- |
| Olanzapine LAI | 1.1-<1.4 | 0.05 (0.01-0.16) |
| Olanzapine LAI | 1.4-<1.6 | 0.07 (0.03-0.19) |
| Olanzapine LAI | ≥1.6 | 0.17 (0.11-0.26) |
| Zuclopenthixol | 0.9-<1.1 | 0.22 (0.03-1.78) |
| Zuclopenthixol LAI | <0.6 | 0.24 (0.16-0.37) |
| Haloperidol LAI | 0.9-<1.1 | 0.28 (0.15-0.53) |
| Perphenazine LAI | 1.1-<1.4 | 0.28 (0.17-0.46) |
| Risperidone LAI | 0.9-<1.1 | 0.28 (0.21-0.39) |
| Zuclopenthixol LAI | 0.6-<0.9 | 0.30 (0.20-0.45) |
| Zuclopenthixol | 1.1-<1.4 | 0.31 (0.03-2.99) |
| Haloperidol | 0.9-<1.1 | 0.32 (0.10-1.07) |
| Perphenazine LAI | <0.6 | 0.32 (0.22-0.48) |
| Haloperidol LAI | 1.4-<1.6 | 0.33 (0.08-1.43) |
| Zuclopenthixol LAI | 0.9-<1.1 | 0.33 (0.22-0.48) |
| Olanzapine LAI | 0.9-<1.1 | 0.34 (0.13-0.88) |
| Risperidone LAI | 0.6-<0.9 | 0.34 (0.27-0.44) |
| Olanzapine | 0.9-<1.1 | 0.35 (0.30-0.42) |
| Clozapine | 1.1-<1.4 | 0.35 (0.31-0.40) |
| Haloperidol LAI | 1.1-<1.4 | 0.36 (0.18-0.71) |
| Zuclopenthixol LAI | 1.4-<1.6 | 0.36 (0.18-0.71) |
| Risperidone LAI | <0.6 | 0.36 (0.24-0.54) |
| Clozapine | 0.6-<0.9 | 0.36 (0.31-0.41) |
| Clozapine | 0.9-<1.1 | 0.36 (0.31-0.42) |
| Perphenazine LAI | 0.6-<0.9 | 0.38 (0.27-0.53) |
| Perphenazine LAI | 0.9-<1.1 | 0.39 (0.24-0.63) |
| Risperidone LAI | 1.1-<1.4 | 0.41 (0.32-0.52) |
| Zuclopenthixol | 0.6-<0.9 | 0.42 (0.17-1.05) |
| Perphenazine LAI | ≥1.6 | 0.42 (0.27-0.65) |
| Clozapine | ≥1.6 | 0.45 (0.40-0.51) |
| Clozapine | 1.4-<1.6 | 0.46 (0.38-0.55) |
| Olanzapine | 1.4-<1.6 | 0.46 (0.39-0.55) |
| Haloperidol | 1.1-<1.4 | 0.47 (0.14-1.55) |
| Aripiprazole | <0.6 | 0.47 (0.32-0.70) |
| Risperidone | 0.6-<0.9 | 0.47 (0.40-0.56) |
| Aripiprazole | 0.9-<1.1 | 0.49 (0.34-0.71) |
| Olanzapine | <0.6 | 0.49 (0.41-0.58) |
| Zuclopenthixol | <0.6 | 0.50 (0.28-0.87) |
| Olanzapine | 1.1-<1.4 | 0.50 (0.43-0.59) |
| Zuclopenthixol LAI | 1.1-<1.4 | 0.51 (0.34-0.76) |
| Clozapine | <0.6 | 0.51 (0.44-0.59) |
| Quetiapine | 0.6-<0.9 | 0.54 (0.44-0.67) |
| Olanzapine | 0.6-<0.9 | 0.54 (0.47-0.62) |
| Haloperidol LAI | <0.6 | 0.55 (0.28-1.08) |
| Chlorprotixene | <0.6 | 0.55 (0.39-0.77) |
| Quetiapine | 1.1-<1.4 | 0.55 (0.44-0.69) |
| Haloperidol LAI | 0.6-<0.9 | 0.56 (0.29-1.07) |
| Risperidone | <0.6 | 0.56 (0.49-0.64) |
| Risperidone | 0.9-<1.1 | 0.57 (0.43-0.77) |
| Olanzapine | ≥1.6 | 0.58 (0.53-0.64) |
| Chlorprotixene | 1.4-<1.6 | 0.60 (0.13-2.67) |
| Chlorprotixene | 0.6-<0.9 | 0.60 (0.27-1.31) |
| Perphenazine LAI | 1.4-<1.6 | 0.60 (0.35-1.02) |
| Aripiprazole | 1.4-<1.6 | 0.61 (0.36-1.05) |
| Quetiapine | 0.9-<1.1 | 0.62 (0.49-0.78) |
| Aripiprazole | 0.6-<0.9 | 0.63 (0.45-0.89) |
| Risperidone LAI | 1.4-<1.6 | 0.64 (0.40-1.05) |
| Aripiprazole | ≥1.6 | 0.65 (0.50-0.85) |
| Risperidone | 1.1-<1.4 | 0.68 (0.52-0.90) |
| Olanzapine LAI | 0.6-<0.9 | 0.69 (0.23-2.04) |
| Quetiapine | <0.6 | 0.70 (0.61-0.80) |
| Perphenazine | <0.6 | 0.72 (0.61-0.85) |
| Chlorprotixene | ≥1.6 | 0.73 (0.29-1.84) |
| Haloperidol | <0.6 | 0.73 (0.54-0.98) |
| Perphenazine | 0.6-<0.9 | 0.74 (0.48-1.14) |
| Perphenazine | 1.4-<1.6 | 0.78 (0.07-8.67) |
| Haloperidol | 0.6-<0.9 | 0.79 (0.45-1.40) |
| Zuclopenthixol LAI | ≥1.6 | 0.79 (0.57-1.09) |
| Aripiprazole | 1.1-<1.4 | 0.82 (0.57-1.17) |
| Quetiapine | 1.4-<1.6 | 0.84 (0.64-1.11) |
| Levomepromazine | <0.6 | 0.85 (0.66-1.09) |
| Haloperidol LAI | ≥1.6 | 0.91 (0.57-1.46) |
| Quetiapine | ≥1.6 | 0.97 (0.82-1.14) |
| Chlorprotixene | 1.1-<1.4 | 1.17 (0.52-2.64) |
| Risperidone LAI | ≥1.6 | 1.24 (0.93-1.66) |
| Risperidone | 1.4-<1.6 | 1.25 (0.77-2.04) |
| Zuclopenthixol | ≥1.6 | 1.37 (0.37-5.09) |
| Perphenazine | 0.9-<1.1 | 1.54 (0.59-4.02) |
| Chlorprotixene | 0.9-<1.1 | 1.64 (0.72-3.77) |
| Risperidone | ≥1.6 | 2.00 (1.46-2.73) |
| Haloperidol | ≥1.6 | 2.49 (1.03-6.02) |
| Perphenazine | 1.1-<1.4 | 2.74 (1.03-7.27) |
| Perphenazine | ≥1.6 | 6.12 (1.17-32.02) |
| Haloperidol | 1.4-<1.6 | NA |
| Levomepromazine | 0.6-<0.9 | NA |
| Levomepromazine | 0.9-<1.1 | NA |
| Levomepromazine | 1.1-<1.4 | NA |
| Levomepromazine | 1.4-<1.6 | NA |
| Levomepromazine | ≥1.6 | NA |
| Olanzapine LAI | <0.6 | NA |
| Zuclopenthixol | 1.4-<1.6 | NA |

**Supplementary Figure 1.** Study design and time-varying exposure.

**
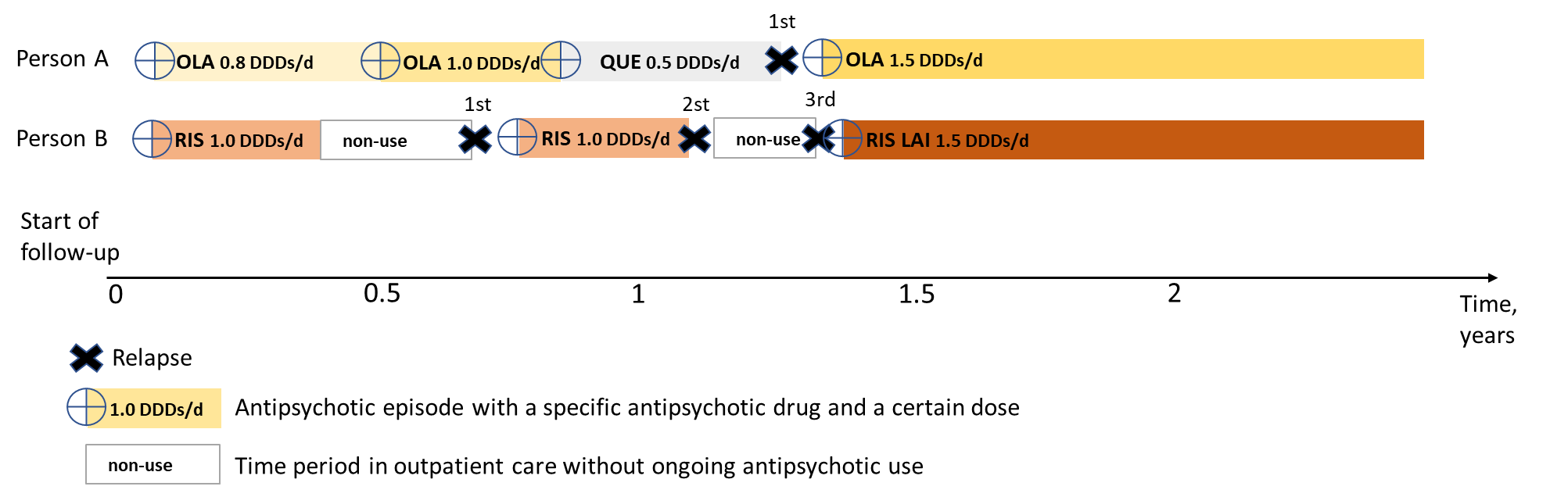
**

**Person A** uses oral olanzapine (OLA) with dose of 0.8 DDDs/day from the start of follow-up up until 0.5 years when olanzapine dose is increased to 1.0 DDDs/day. About 0.8 years after start of follow-up olanzapine is switched to quetiapine (QUE) which is used 0.5 DDDs/day. Person has his/her first relapse in the study at timepoint 1.25 years during quetiapine use. After discharged from the first relapse, person is switched back to oral olanzapine which is now dosed 1.5 DDDs/day. In time-varying Cox model, Person A has four dose periods and contributes to the analyses of both olanzapine and quetiapine. Doses are categorized as olanzapine 0.7-<0.9 DDDs/day, olanzapine 0.9-<1.1 DDDs/day, quetiapine <0.6 DDDs/day and olanzapine 1.4-<1.6 DDDs/day, respectively.

**Person B** uses first oral risperidone (RIS) at dose 1.0 DDDs/day and about 0.4 months after start of follow-up discontinues the use. Person B has his/her first relapse at timepoint 0.7 months after start of follow-up during non-use of antipsychotics. After first relapse, person is back with risperidone 1.0 DDDs/day and has second relapse during it’s use. After second relapse, person is not using antipsychotics and has third relapse during non-use. After third relapse, person is using risperidone long-acting injectable (LAI) at 1.5 DDDs/day until end of this illustration. In time-varying Cox model, Person B has three dose periods (of which two first are the same risperidone 1.0 DDDs/day dose, categorized as 0.9-<1.1 DDD/day, and one with risperidone LAI 1.4-<1.6 DDDs/day), two non-use periods and this person contributes to the analyses of oral risperidone and risperidone LAI.
